# Total chemical synthesis of photoactivatable proteins for light-controlled manipulation of antigen–antibody interactions†
†Electronic supplementary information (ESI) available: Experimental section. See DOI: 10.1039/c5sc03404c


**DOI:** 10.1039/c5sc03404c

**Published:** 2015-12-11

**Authors:** Shan Tang, Zhengpeng Wan, Yiren Gao, Ji-Shen Zheng, Jing Wang, Yan-Yan Si, Xin Chen, Hai Qi, Lei Liu, Wanli Liu

**Affiliations:** a Tsinghua-Peking Center for Life Sciences , Key Laboratory of Bioorganic Phosphorus Chemistry & Chemical Biology (Ministry of Education) , Department of Chemistry , Tsinghua University , Beijing 100084 , China . Email: lliu@mail.tsinghua.edu.cn; b MOE Key Laboratory of Protein Science , Collaborative Innovation Center for Diagnosis and Treatment of Infectious Diseases , School of Life Sciences , Tsinghua University , Beijing , 100084 , China . Email: liulab@mail.tsinghua.edu.cn; c High Magnetic Field Laboratory , Chinese Academy of Sciences , Hefei , 230031 , China; d Laboratory of Dynamic Immunobiology , School of Medicine , Tsinghua University , Beijing , 100084 , China

## Abstract

We report the chemical synthesis of the first photo-activatable protein antigen that can be used to study antigen–antibody interaction mediated responses in B cells. This strategy facilitated fine tuning of the caged protein antigen to optimize its bioactivity and photochemical properties. One optimal molecule, HEL-K_96_NPE, was totally inert to hen egg lysozyme (HEL)-specific B cells and could only restore its antigenicity upon photoactivation. Combined with real time live cell imaging, the utility of HEL-K_96_NPE was demonstrated as a proof of concept to quantify B cell synapse formation and calcium influx responses at the single cell level.

## Abstract 2

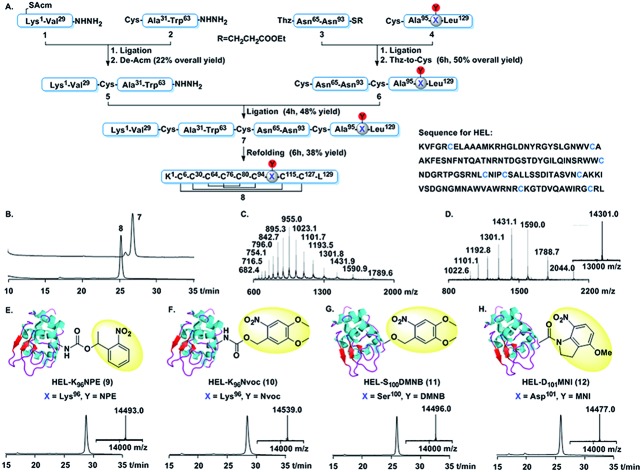
We report the chemical synthesis of the first photo-activatable protein antigen that can be used to study antigen–antibody interaction mediated responses in B cells.

## 

Light-responsive reagents provide useful tools to probe and manipulate biological events in a controllable manner with an aim to elucidate complex biological processes.^1^ Since the report of caged cAMP and ATP,^2^ various caged biomolecules such as neurotransmitters,^3^ nucleotides,^4^ lipids,^5^ and peptides^6^ have been described. Photocaged proteins,^7^ due to their high bioactivity and specificity, are especially suitable for the studies of dynamic cellular processes such as signal transduction, DNA transcription and cell motility.^8^ Photocaged proteins are typically obtained by masking the critical functionality for enzyme activity/protein–protein interaction or peptide backbones.^9^ Although the technique of genetically encoding unnatural amino acids makes the preparation of photocaged proteins possible,^10^ chemical protein synthesis can also be used for development of photocaged proteins.^11^

Here we report the first photoactivatable protein antigen developed through chemical protein synthesis for light-controlled manipulation of antigen–antibody interactions in B cell studies. B lymphocytes circulating in the blood and lymphatic system perform important roles of immune surveillance. They use the surface expressed B cell receptors (BCRs) composed of membrane bound antibody and signaling co-receptor Igα/Igβ to recognize and capture pathological antigens.^12^,^13^ Much attention has been paid to the dynamic events driving the formation of the B cell immunological synapse (IS) using imaging approaches.^14^ A better understanding of these events provides insights into fundamental aspects of B cell responses, such as antibody discrimination, antibody memory immunity and even B cell tumorigenesis.^15^ Nonetheless, there is a lack of molecular tools to manipulate the initiation of these dynamic events in a controllable manner.^16^ Our caged protein antigens provided novel and useful tools to cope with this challenge.

We focused on hen egg lysozyme (HEL), one of the most important model antigens recognized by the B cells expressing HEL-specific membrane bound antibody (HyHEL-10).^17^ Studies on HEL have greatly contributed to the understanding of antibody responses and B cell biology.^18^,^19^ An inert caged antigen (Scheme 1A) that could only be activated upon photoactivation would be useful to study antigen–antibody interactions and the formation of B cell IS in a controllable manner. This created an interesting challenge as the HEL/HyHEL-10 interaction is very strong (*K*_D_ ≈ 20 pM) with a very large interaction surface (about 1800 Å) (Scheme 1B).^20^,^21^ Among the 16 residues directly contacting the HyHEL-10 antibody, the external surface of the helix and the extended loop region (Thr^89^–Gly^102^, Scheme 1C) contributes the most to the interaction of HEL with the HyHEL-10 antibody. To obtain a photoactivatable HEL, we decided to mask key amino acid residues lying on the interface based on the co-crystal structure of HEL and Fv fragment of HyHEL-10 antibody.^21^

**Scheme 1:**
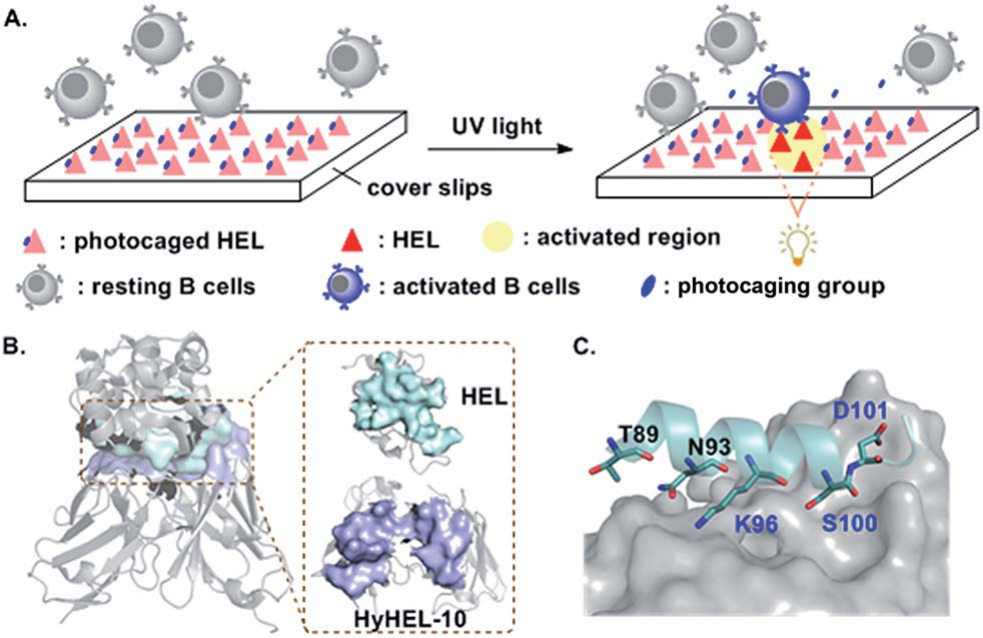
(A) Photocaged HEL, after exposure to near-UV light, activates B cells to trigger downstream signal transduction. (B) Co-crystal structure of HEL and Fv fragment of HyHEL-10 (left). The interface of HEL and HyHEL-10 is shown on the right. (C) Interaction between the external surfaces of HEL and HyHEL-10. The images are modified from Protein Data Bank file 2DQJ.

We first developed a general synthetic route to HEL. An earlier synthesis of human lysozyme used *tert*-butoxycarbonyl (Boc) solid phase peptide synthesis (SPPS).^22^ However, as the caging groups were not compatible with Boc SPPS,^23^ a 9-fluorenyl-methoxycarbonyl (Fmoc) route was needed.^24^ As shown in Fig. 1A, HEL was divided into two halves, each containing two segments, *i.e.* (**1**, **2**, **3** and **4**). Cys^6^ in segment **1** was protected by an acetamidomethyl (Acm) group to avoid the formation of thiolactone.24*c* The N-terminal half **5** was assembled by ligation of **1** and **2**, followed by removal of the Acm group. The C-terminal half **6** was made by condensation of **3** and **4**, and subsequent conversion of Thz to Cys. Final ligation between **5** and **6** afforded full-length HEL (**7**), which was subjected to a redox system to form the correctly folded HEL (**8**). **7** and **8** were characterized by HPLC and mass analysis (Fig. 1B–D). The pattern of four disulfide linkages was consistent with that of native HEL according to (LC-MS)/MS analysis (Fig. S14 and S15†).

**Fig. 1:**
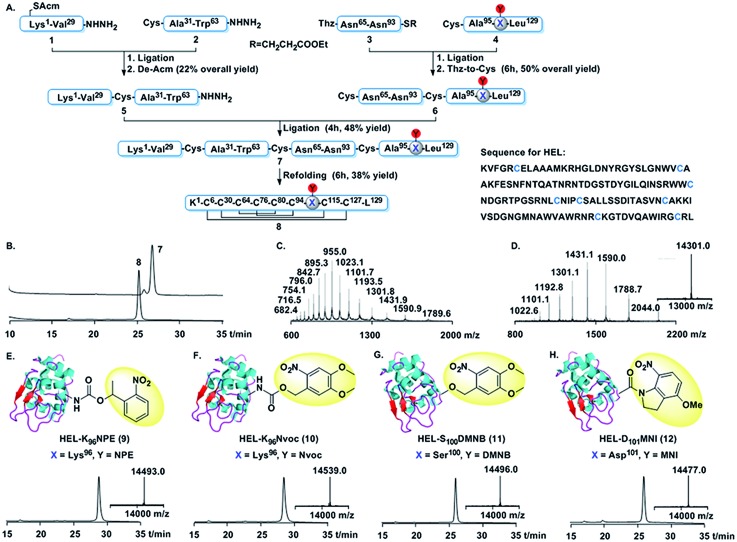
Chemical synthesis of HEL derivatives. (A) Synthetic route. (B) HPLC traces (214 nm) for full-length and folded HEL. (C) ESI-MS of full-length HEL. Observed mass = 14 309.2 ± 0.8 Da (cacld 14 308.9 Da, average isotopes). (D) ESI-MS and deconvoluted mass of HEL. Observed mass = 14 301.3 ± 0.7 Da (cacld 14 300.9 Da, average isotopes). (E–H) Chemical structure, HPLC traces (214 nm) and deconvoluted mass (inner) of **9** (observed mass = 14 493.0 Da, cacld 14 494 Da), **10** (observed mass = 14 539.0 Da, cacld 14 540 Da), **11** (observed mass = 14496.0 Da, cacld 14 496 Da), and **12** (observed mass = 14 477.0 Da, cacld 14 477 Da).

Using the above synthetic route, we synthesized the photocaged HELs (Fig. 1E–H). We aimed to develop near UV-sensitive proteins for *in vitro* live cell imaging studies. Therefore, we used 1-(2-nitrophenyl)ethyl (NPE) and 6-nitroveratryloxy-carbonyl (Nvoc) groups to mask Lys^96^, obtaining HEL-K_96_NPE (**9**) and HEL-K_96_Nvoc (**10**). We also made HEL-S_100_DMNB (**11**) using 4,5-dimethoxy-2-nitrobenzyl (DMNB) to mask Ser^100^. In addition, we made HEL-D_101_MNI (**12**) using 4-methoxy-7-nitroindolinyl (MNI) to mask Asp^101^.^25^ HPLC analysis showed that all four photocaged HEL variants exhibited enhanced hydrophobic features compared to HEL, reflecting the hydrophobic nature of each photocleavable moiety. Each HEL variant was obtained at the 1 mg scale (Fig. S4–S12, Table S2†).

CD spectra of these photocaged proteins and native HEL exhibited a negative minimum absorption at 208 nm,^26^ indicating that attachment of photosensitive moieties did not appreciably change the general structure of HEL (Fig. S16†).

We examined the antigenicity of the synthetic proteins against HyHEL-10 with an antigen-dose dependent enzyme-linked immunosorbent assay (ELISA). Synthetic wild-type HEL exhibited a binding capacity similar to that of native HEL purified from hen egg white (Fig. S17†), while **9** and **10** exhibited no measurable binding to HyHEL-10 (Fig. 2A). Moreover, the binding ability of **11** or **12** was reduced but not abrogated. A similar conclusion was made after measuring the binding affinity of the HEL variants to HyHEL-10 by surface plasmon resonance (SPR) (Fig. S20†). The binding affinity of **12**, **11** and **9** (or **10**) to HyHEL-10 was about 300-, 400-, and 6000-fold weaker than that of native HEL. Therefore, caging at Lys^96^ (**9** or **10**) can effectively reduce the binding affinity of HEL toward HyHEL-10. Upon exposure to UV light (365 nm, 18 mW cm^–2^) for 60 s, we observed enhanced binding capability to HyHEL-10 for all of these four photocaged HEL variants (Fig. 2B). Especially for **9**, the binding capability after photoactivation was comparable to native HEL. However, **10** only partially restored its antigenicity. These results were consistent with the photolysis kinetics data obtained by analyzing the percentage of photolytic peptides in the total peptide population (Fig. 2C and S19†).^27^

**Fig. 2:**
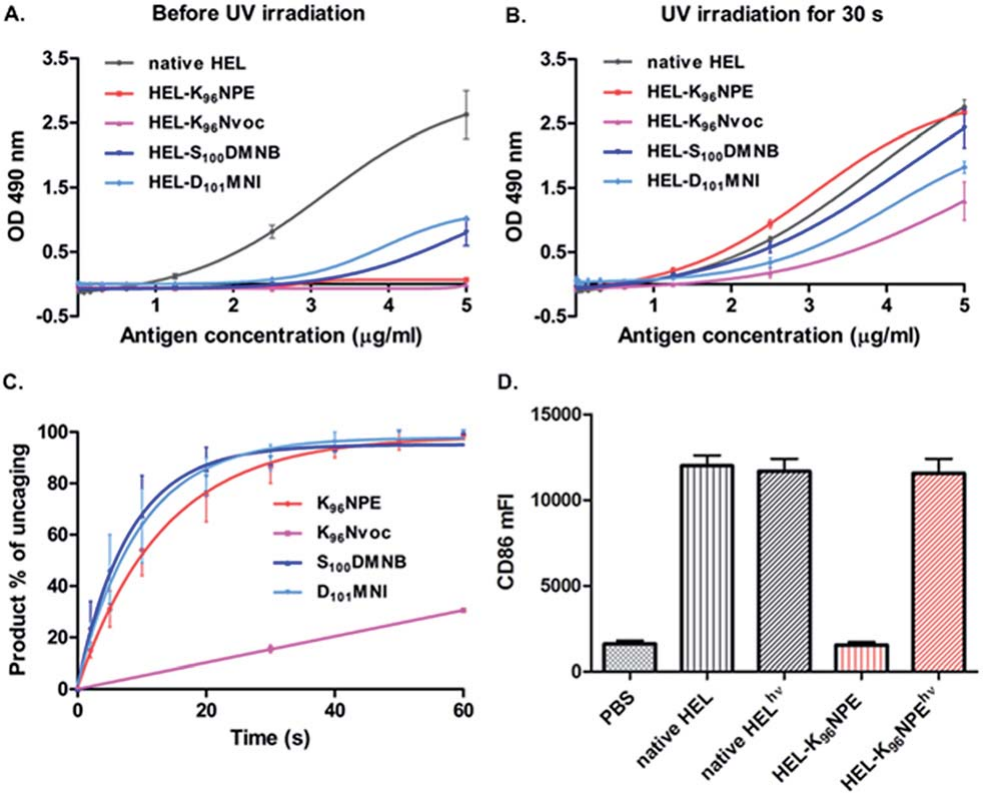
Photocaged HEL-K_96_NPE restored its antigenicity upon irradiation. The binding capacities of HEL variants to HyHEL-10 were measured by ELISA before (A) and after (B) exposure to UV light for 60 s. (C) Photolysis kinetics for caged HEL segments. (D) Photoactivated HEL-K_96_NPE efficiently increased the CD86 expression in MD4 primary B cells.

To further assess the antigenicity of photoactivated **9** in living B cells, a flow cytometry-based cellular assay was carried out. Photoactivated **9** efficiently up-regulated the activation marker CD86 on HEL-specific primary B cells from MD4 transgenic mice (MD4 primary B cells) similarly to native HEL,^28^ while **9** was totally inert after an incubation duration of 12 h (Fig. 2D and S21†). These results showed that caging of a single amino acid can efficiently disrupt a picomolar affinity protein–protein interaction with a large interface up to 1800 Å^2^.

We then used **9** to temporally control antigen–antibody mediated B cell spreading and the subsequent accumulation responses of BCRs into the B cell IS.^14^ We immobilized **9** on the surface of cover slides, and placed freshly isolated MD4 primary B cells (pre-labeled with Alexa Fluor 647 conjugated Fab fragment of anti-mouse IgM) on these slides. The dynamics of the contact interface of B cells with glass slides before and after irradiation with a 405 nm laser (7.3 W cm^–2^) were examined by TIRF imaging.^29^,^30^ As shown from the images (Fig. 3A and ESI Movie 1†), before laser irradiation, MD4 primary B cells mildly touched the cover slides coated with **9**. Only 3 s after photoactivation, MD4 primary B cells underwent a typical spreading response towards the photoactivated surface as quantified by the increased size of the B cell contact area, which was referred to as the size of B cell IS (Fig. S29†). The mean fluorescence intensity (mFI) of HyHEL-10 BCRs within the B cell IS increased by about 400% in 400 s after photoactivation (Fig. 3B). These responses were dependent on the light dosage in a linear manner (Fig. S27†) and only B cells within the photoactivated region but not the B cells sitting far beyond the photoactivated region exhibited the synaptic accumulation of BCRs (Fig. S26†). These data indicated that immediately after photoactivation, **9** was converted to the antigenic form to drive the accumulation of BCRs at the photoactivated regions.

**Fig. 3:**
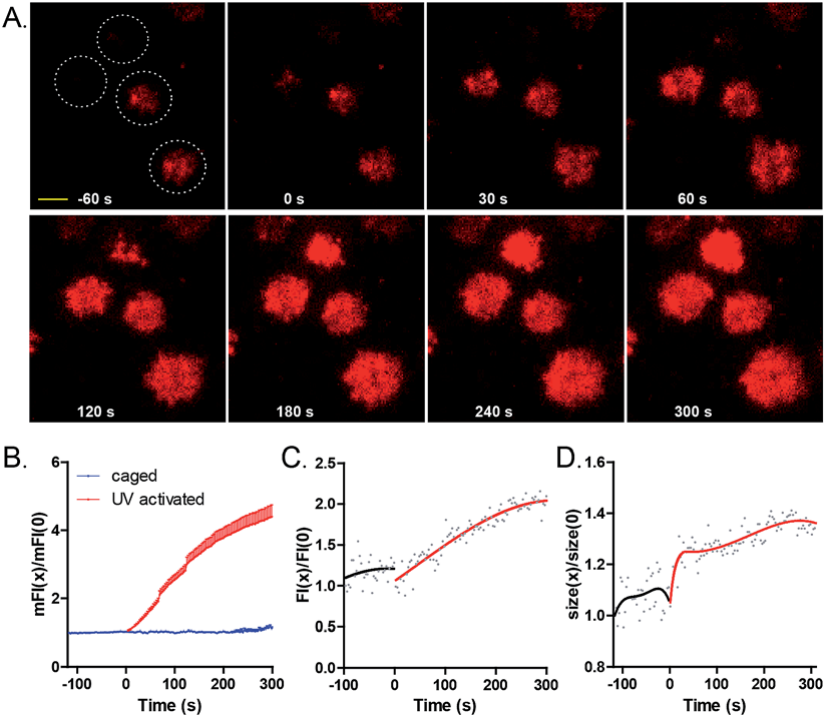
Photoactivation of **9** by a 405 nm laser efficiently drove B cell spreading responses and the accumulation of BCRs into the B cells IS. (A) Representative TIRF images of MD4 primary B cells encountering **9** before and after photoactivation. Four independent cells are indicated by the white colored dashed circles. Scale bar, 1.5 μm. Statistical quantification of (B) the mean fluorescence intensity (FI) of BCR within the IS, (C) FI and (D) size of BCR microclusters before and after photoactivation. Normalized data was collected from 41 cells for the activated group and 11 cells for the inactivated group.

Next we examined the dynamic changes of the high BCR FI puncta structures (BCR microclusters) within the contact interface of B cells with cover slides before and after photoactivation. As shown by the four representative cells in Fig. 3A, there was no formation of stable and prominent BCR microclusters before photoactivation. However, statistical analysis showed that the BCR microcluster FI and size (Fig. 3C and D), as quantified by a 2D-Gaussian function-based fitting method,^31^ increased over time after photoactivation. These results suggested that BCR and antigen recognition triggered the formation and maturation of BCR microclusters during the initiation of B cell activation.14*a*

Finally, we quantified the single cell calcium influx responses upon photoactivation. MD4 primary B cells pre-stained with the calcium probe Fluo-4 were placed on cover slides presenting **9** for calcium imaging (Fig. 4A and ESI Movie 2†). No calcium influx responses were observed in the MD4 primary B cells in contact with **9** in the first 400 s. Upon photoactivation (405 nm laser, 7.3 W cm^–2^), uncaged **9** readily drove the B cells to undergo calcium influx responses. The oscillating calcium influx responses were observed with an oscillation cycle of 85–90 s and an amplitude of 2–2.6 times the basal mFI level (Fig. 4B, and ESI Movie 2†). The oscillating calcium influx nature is the physiological response of activated primary B cells *in vivo*.^32^ Thus with the help of the photoactivatable HEL, we can capture the full frame dynamic calcium influx responses in the initiation of B cell activation that are not easily observed by the conventional experimental system.

**Fig. 4:**
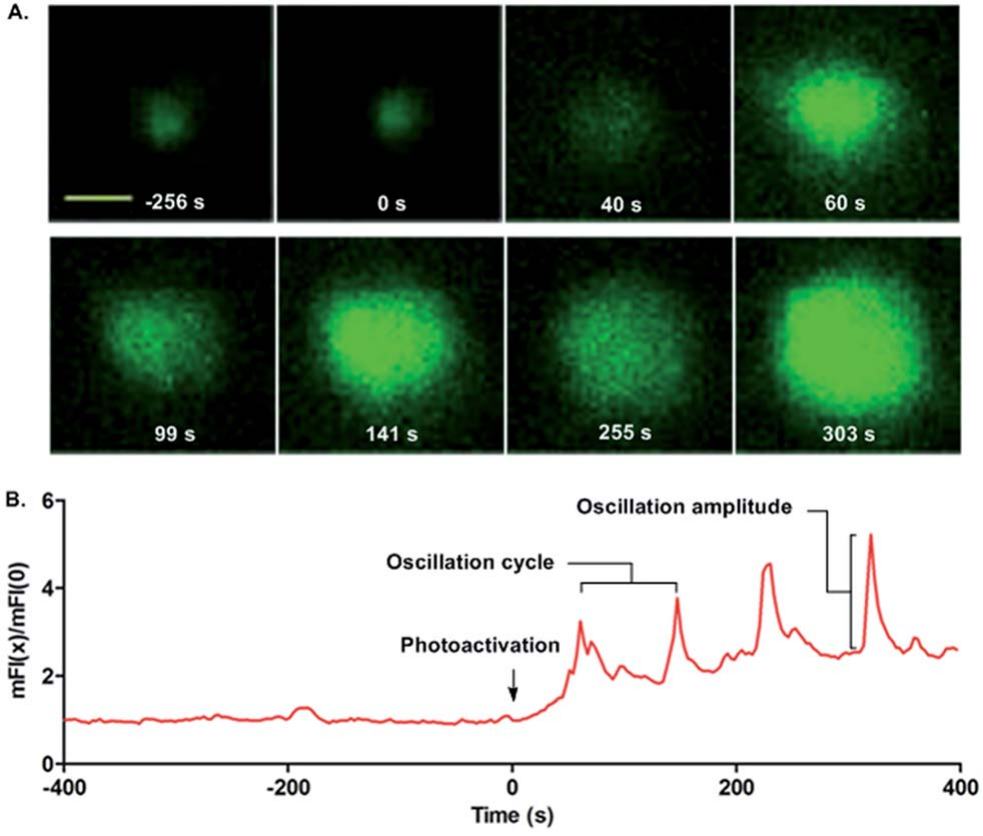
Photoactivated HEL-K_96_NPE induced calcium signaling at the single cell level. (A) Fluorescence images and (B) normalized mean fluorescence intensity of single cell calcium oscillation. Scale bar, 3 μm.

It should be pointed out that although **9** failed to activate HEL-specific BCR and B cells, it showed a binding affinity to HyHEL-10 of about 50 nM in our SPR assay (Fig. S20†). Because the affinity between wild type HEL and HyHEL-10 test in our SPR assay was 2–7 fold higher than that was measured by other methods in the literature,^19^,^20^,^33^ we cannot completely rule out the possibility that we might have also overestimated the affinity between HyHEL-10 with HEL-K_96_NPE. However, previous studies showed that HEL(K97A) mutant with an affinity of 114 nM to HyHEL-10, exhibiting a 2400-fold decrease to HEL, lost the ability to activate B cells.^19^ Based on these results, we proposed that in addition to binding affinity, the availability of a sufficient amount of antigen density,^19^ a high-enough *K*_on_ rate,^19^ and even the correct conformational changes upon antigen binding^34^ might be cooperating in the binding for the conversion to constructive biological effects.

## Conclusions

Protein chemical synthesis offers convenient access to tailor-designed photocaged proteins. We developed the first photoactivatable protein antigen through photocaged hot spot scanning. The *in vitro* ELISA- and SPR-based binding assays and flow cytometry-based cellular assays provided the basis for subsequent studies investigating the dynamic early events by TIRFM-based live cell imaging approaches. Importantly, we captured the behavior changes of B cells upon photoactivation for spreading responses, the accumulation of BCR molecules into the B cell immunological synapse and the calcium oscillation response in a controllable manner. By offering the unique ability to trigger the antigen–antibody interaction with an exact temporal resolution, HEL-K_96_NPE could be a promising molecular tool to unravel the complex and dynamic events in the initiation of B cell activation. Our results also indicate the powerful capability of chemical protein synthesis in enabling the optimization of bioactivities and photochemical properties of caged proteins.

## Supplementary Material

Supplementary information

Supplementary movie

Supplementary movie
