# Supplementary material for: Total chemical synthesis of photoactivatable proteins for light-controlled manipulation of antigen–antibody interactions
Source: Chem Sci. 2015 Dec 11;7(3):1891–5. doi: 10.1039/c5sc03404c (PMC5965250; doi:10.1039/c5sc03404c)
Supplement: Supplementary file 1 [file SC-007-C5SC03404C-s001.pdf]

Electronic Supplementary Material (ESI) for Chemical Science.
This journal is © The Royal Society of Chemistry 2015

Supporting Information

Table of Contents

Methods:

General methods ••S2

Supporting text and Figures:

Part I: Synthesis of photocaged amino acid building blocks ••••••••••••••••••••••••••S7

Part II: Synthesis of HEL and its derivatives •••S14

Part III: Characterization of the antigenicity of HEL variants ••••••••••••••••••S31

Part IV: Molecular imaging by TIRFM to quantify the formation of B cell immunological synapse •••S36

Part V: Supporting movie legends ••S42

Supporting References ••S43

General Methods

Peptide synthesis. All peptides were synthesized using Fmoc-based strategy according to the standard protocol.[1] The resin was coupled with amino acid (4 eq.), coupling reagent (3.6 eq. HATU or HCTU) in 0.25 M DIEA solution. Photocaged amino acids were coupled to the resin using the DIC/HOBt method. 2 eq. caged amino acid, 5 eq. DIC and 5 eq. HOBt was recommended to react with the resin overnight. Ninhydrin test was used to monitor the coupling reaction. After the assembly completed, peptides were cleaved from resin by TFA cocktail (88% TFA, 5% H_2O, 5% Phenol, 2% TIPS), and then analyzed and purified by reversed-phase HPLC (CH_3CN-H_2O, 0.1% TFA), molecular weight of each peptide was confirmed by electrospray ionization (ESI) mass spectrometry (MS).

Reversed-Phase HPLC (RP-HPLC). Analytical and semi-preparative RP-HPLC were performed with a Prominence LC-20AT with SPD-20A UV/Vis detector. A Vydac C18 or C4 column (5 μm, 4.6 mm×250 mm) was used for analytical RP-HPLC with a 1 mL/min flow rate, and Vydac C18 or C4 columns (10 μm, 10×250 mm) with a 4 mL/min flow rate were used for semi-preparative RP-HPLC with buffer A (0.1% TFA in water) and buffer B (0.1% TFA in CH_3CN). Gradients for analyzing all peptides were detailed below. Data were recorded and analyzed using the software system LC Solution.

Mass Spectrometry. Target containing fractions were identified by ESI-MS. ESI-MS was performed on an Agilent 1200/6340 mass spectrometer in Center of Biomedical Analysis (Tsinghua University). 50% CH_3CN/H_2O (v/v) containing 0.1% formic acid was used as buffers for LC/MS analysis to increase MS ionization of peptides.

LC-MS/MS analysis. HEL reduced with 10 mM of DTT and alkylated with 55 mM iodoacetamide or directly digested by the sequence grade modified trypsin (Promega, Fitchburg, WI) in 50 mM ammonium bicarbonate at 37 °C overnight. The peptide fragments were extracted twice with 50% CH_3CN/H_2O (v/v) containing 1% TFA for 30 min. The extractions were then concentrated and separated by a 65 min gradient elution at a flow rate of 0.25 µL/min with a C18 column. The EASY-nLCIITM integrated nano-HPLC system (Proxeon, Denmark), which was directly interfaced with the Thermo LTQ-Orbitrap mass spectrometer, was used for LC-MS/MS analysis. The LTQ-Orbitrap mass spectrometer was operated in the data-dependent acquisition mode using the Xcalibur 2.0.7 software. The MS/MS spectra from each LC-MS/MS were searched against the database using an in-house Mascot or Proteome Discovery searching algorithm.

Chemical ligation of peptide hydrazides. Peptide hydrazides were dissolved in 0.2 M aqueous phosphate buffer containing 6.0 M Gn·HCl at pH 3.0 and cooled to -15 ^{o}C. 10 equiv. $NaNO_2$ was added to the solution, which was gently agitated for 15 min. Subsequently, MPAA (final concentration of 0.2 M) and 1.0 equiv. N-terminal Cys-peptide was added to the reaction mixture, followed by pH adjustment to 6.5 to conduct the ligation reaction. The ligation was monitored by analytical HPLC and ESI-MS. When the ligation went to completion, 0.1 M neutral TCEP solution was added to the reaction mixture for 20 min to reduce the ligation system, which was

then purified by preparative HPLC.

Chemical ligation of Thz-protected peptide thioester and N-terminal Cys-peptide. Equal equivalent of Thz-protected peptide thioester and N-terminal Cys-peptide (2 mM) were dissolved in 0.2 M aqueous phosphate buffer containing 6.0 M Gn·HCl, 0.2 M MPAA, 0.1 M TCEP at pH 6.5. When the ligation completed, solid $MeONH_2$·HCl was added to the solution to reach the concentration of 0.25 M, followed by pH adjustment to 4 to convert Thz to Cys. The whole process was monitored by analytical HPLC and ESI-MS. The desired ligated N-terminal Cys-peptide was purified by preparative HPLC. When the N-terminal Cys-peptide contained photosensitive moieties, $MeONH_2$·HCl was recommended to be dissolved in equal volume of 0.2 M aqueous phosphate buffer containing 6.0 M Gn·HCl. Addition of $MeONH_2$ containing solution was effectively to prevent precipitation formation.

Circular dichroism (CD) spectroscopy. CD spectra were recorded on a Pistar π-180 spectrometer from 260 nm to 190 nm at 25 °C in a quartz cell with 1 mm path length. The final concentration of HEL, HEL-K_{96}NPE, HEL-K_{96}Nvoc, HEL-S_{100}DMNB and HEL-D_{101}MNI was about 10 μM. The spectra were performed in triplicate, averaged, subtracted from blank and smoothed.

Photochemistry. Caged peptides or proteins (20-50 μL) in PBS buffer (pH 7.4) were placed in a 1.5 mL eppendorf tube kept on ice. Irradiation by UV light (365 nm) was performed using Omnicure S1500 (EXFO Photonic Solutions Inc., Canada) with the light intensity of 18 mW/cm^2. RP-HPLC analysis was used to determine the photolysis kinetics. Peptides were quantified based on the HPLC standard curves of the caged and uncaged derivatives. The amount of each peptide was normalized to the amount of Benzamide as the internal standard.

Surface plasmon resonance (SPR) experiments. The affinities between HyHEL-10 and different versions of HEL were measured by SPR (Biacore T200, GE Healthcare, Sweden) at 25 °C. HyHEL-10 antibody (0.5 mg/mL) was diluted with 10 mM sodium acetate buffer (pH 4.5, GE Healthcare) to reach the final concentration of 30 μg/mL, which was covalently immobilized to flow cell 2 of a CM5 sensor chip (GE Healthcare) via the standard Amine Coupling Kit (GE Healthcare). The target HyHEL-10 immobilization level was 700 Response Unit (RU). The flow cell 1 was blank as a reference, without any modification. Measurements were run at a flow rate of 30 μL/min in HBS-P buffer (0.1 M HEPES, 1.5 mM NaCl, 30 mM EDTA, 0.5% surfactant P20, pH 7.4, GE Healthcare). A concentration gradient of HEL, HEL-K_{96}NPE, HEL-K_{96}Nvoc, HEL-S_{100}DMNB, and HEL-D_{101}MNI was freshly prepared in running buffer with at least five concentrations, which were optimized according to different analytes. The analytes at non-zero concentrations (the middle concentration was repeated) and one zero concentration (running buffer) were injected over the chip at a flow rate of 30 μL/min, with the contact time of 180 s and dissociation time of 600 s for HEL, HEL-K_{96}NPE, HEL-K_{96}Nvoc, HEL-$S1_{00}$DMNB and 300 s for HEL-D_{101}MNI. After each injection of analytes, the sensor chip's surface was regenerated with 10 mM Glycine 1.5 (GE Healthcare) at a flow rate of 30 μL/min for 60 s to completely remove the tightly-bound analytes. All of the experimental data were analyzed by kinetic model using Biacore T200 Evaluation Software, version 1.0. The binding affinity of each analyte to HyHEL-10

antibody was tested three times and averaged.

Cells, reagents and antibodies. HEL-specific primary B cells were isolated from spleens of C57BL/6 IgHEL-MD4 transgenic mice. Alexa Fluor 647-conjugated Fab fragment Goat anti-mouse IgM (Jackson) was used to stain the surface expressed IgM-BCRs of MD4 B cells. Fluo4-AM (Invitrogen and probenecid Life Technologies) was used to stain the calcium cation in MD4 B cells. Polyclonal HRP conjugated goat anti-mouse IgG was bought from Boster. PE Rat anti-mouse CD19 IgG and APC Rat anti-mouse CD86 IgG were bought from eBioscience.

ELISA. Binding capacities of the commercially bought native HEL, synthetic HEL and four photocaged HEL derivatives before (0 s) and after exposure to UV light (365 nm) for different periods of time (30 s, 60 s, 300 s) were measured according to the standard ELISA methods. Each HEL variant was serially diluted in a fold of two starting from the concentration of 5 μg/mL. Each solution was coated on maxisorb plates (Nunc) and incubated overnight at 4 ^{o}C. Then 0.3% of gelatin in PBS buffer was used for blocking (2 h at 37 ^{o}C), followed by addition of HyHEL-10 antibody at the final concentration of 5 μg/mL into each well and incubated at 37 ^{o}C for 1 h. 1:10,000 diluted HRP conjugated goat anti-mouse IgG was used to detect HyHEL-10 antibody.

Flow cytometry analysis for B cell activation marker CD86. Expression level of the B cell activation marker CD86 from CD19^{+} MD4 primary B cells was analyzed by flow cytometry. MD4 spleen cells were incubated with 100 ng/mL of HEL, HEL-K_{96}NPE, and irradiated (365 nm UV light, 18 mW/cm^2, 60 s) HEL and HEL-K_{96}NPE at 37 ^{o}C for 12 hours, respectively. Before staining, MD4 spleen cells were collected and re-suspended in 100 μL PBS buffer and pre-blocked by FcγRII-blocking antibody 2.4G2 (1 μg/mL, at 10 ^{o}C for 30 min). After that, these cells were stained by PE-conjugated rat anti-mouse CD19 and APC-conjugated rat anti-mouse CD86 at the final concentration of 1 μg/mL. The staining step was performed at 0 ^{o}C for 30 min and washed 3 times with PBS buffer and suspended into 400 μL PBS buffer. In flow cytometry, 30,000 events were collected for each sample. This acquisition was done by the BD LSR II system and data was processed by FlowJo software.

Molecular imaging by Total Internal Reflection Fluorescence Microscope (TIRFM). Molecule images were acquired by an Olympus IX-81 microscope supported by a TIRF port, ANDOR iXon+ DU- 897D electron-multiplying EMCCD camera, Olympus 100 x 1.45 NA objective TIRF lens, a 488 nm, a 561 nm and a 633 nm laser (Sapphire lasers, Coherent). The exposure time was 100 ms except for HEL-K_{96}NPE uncaging (3 s). Acquisition was controlled by Metamorph software (MDS Analytical Technologies). Images were analyzed by Image Pro Plus (Media Cybernetics), Image J (NIH, U.S.), or Matlab (Mathworks) software.

Immobilization of HEL antigen to cover slides. Eight-well chamber slides (Labtak chambers, Thermo Fisher Scientific) were cleaned using a "piranha" solution (70% H_2SO_4 / 30% H_2O_2) at 90 ^{o}C for 1 h, and then washed by H_2O, and dried under N_2. These freshly cleaned eight-well chamber slides were coated with HEL or photocaged HEL variants (100 μL antigen at the concentration of 5 μg/mL in PBS) for 30 min at 37 ^{o}C, washed with PBS, and allowed to dry. The coated slides were washed with PBS and then incubated in 200 μL blocking buffer (0.1% casein in

PBS) for 1 h at 37 ^{o}C. The slides were then washed with PBS were ready for downstream experiments.

Accumulation of BCR into the Immunological Synapse. HEL specific B cells isolated from MD4 mouse spleen were placed on the cover-slide surface pre-coated with HEL or HEL-K_{96}NPE. MD4 primary B cells were pre-incubated with these coated cover-slides at 37 ^{o}C for 10 min before the photoactivation experiments. The formation and accumulation of BCR microclusters into the immunological synapse (IS) were examined before and immediately after photoactivation by TIRFM. Images were taken in a time lapse manner. The exposure time to image BCR was 0.1 s with an interval time of 3 s for photoactivation. The total amount of uncaging time for 405 nm laser is 30 s in TIRFM imaging experiments. The intensity of the 405 nm laser is 7.3 W/cm^2 during photoactivation step, and is 0 W/cm^2 during the TIRF imaging step. The mathematical analyses and quantification of the accumulation of the TIRFM images were processed following our published protocols.[2-4]

Mathematical quantification of BCR microclusters for fluorescence intensity (FI) and size information. Precise two-dimensional integrated FIs of these microclusters were acquired by means of least squares fitting of a two-dimensional Gaussian function. Such a two-dimensional Gaussian function was used to quantify each of the two-dimensional FI profiles, which provided several key parameters for each microcluster including the position (xc, yc), integrated FI (I), background FI (z0), and full width at half-maximum peak height of the intensity distribution.

Calcium oscillation of B cells. Calcium oscillation of MD4 primary B cells was imaged by an epifluorescence microscope in a time lapse manner after loading the stained B cells to the chamber glass slides pre-coated with HEL or HEL-K_{96}NPE. The coated cover-slides were washed with PBS and blocked with 200 μL 0.1% casein at 37 ^{o}C for 50 min. For staining, MD4 B cells were suspended in 500 μL PBS buffer with Fluo4-AM and probenecid at their working concentration (1 μg/mL for Fluo4-AM and 100 μM for probenecid). The cells were incubated at 37 ^{o}C for 30 min and washed with PBS followed by another incubation of 45 min at 37 ^{o}C, and washed twice. After that, the BCRs were stained with 200 nM Alexa Fluor 647-conjugated Fab fragment Goat anti-mouse IgM for 3 min on ice and the cells were washed 3 times with PBS buffer and re-suspended into HBS buffer with 10% FBS. The cells were loaded on the chamber slice and incubated at 37 ^{o}C for 10 min before imaging. The responses of the B cells before and immediately after photoactivation of HEL-K_{96}NPE were examined by TIRFM. Images were analyzed by Image J (NIH, U. S.) and Image Pro Plus software following our published protocols.[2-4]

Chemical synthesis of photocaged amino acids. All reagents and solvents were bought from Sinopharm Chemical Reagent Co. Ltd or Alfa Aesar and were purified if necessary. Anhydrous solvents (THF, CH_2Cl_2, EtOAc) were obtained from a dry solvent system (passed through column of alumina). Thin-layer chromatography (TLC) was performed on silica 60F-254 plates. The spots were visualized by UV light, ninhydrin test, or potassium permanganate staining. Flash column chromatography was performed on silica gel 60 (300-400 mesh). ^{1}H and ^{13}C NMR spectra were recorded on a JOEL 300 or 400 MHz instrument at room temperature in $CDCl_3$. Chemical shifts (δ) were reported relative to TMS (^{1}H 0 ppm) or $CDCl_3$ (^{1}H 7.26 ppm) for ^{1}H-NMR and $CDCl_3$

(77.16 ppm) for ^{13}C-NMR spectra. J was given in Hertz (Hz), and the splitting patterns were designed as follows: s, singlet; bs, broad singlet; d, doublet; t, triplet; m, multiplet.

Part I: Synthesis of photocaged amino acid building blocks

1. Synthesis of Fmoc-Lys(DMNB)-OH.

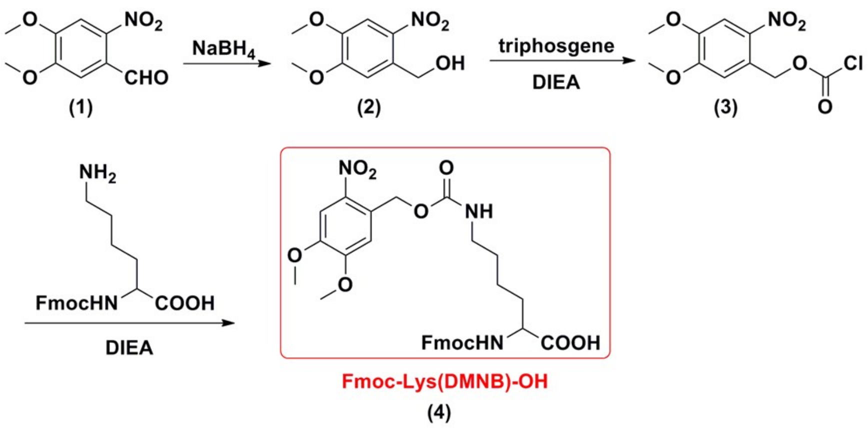

Scheme S1. Synthetic route for Fmoc-Lys(DMNB)-OH.

Fmoc-Lys(DMNB)-OH was synthesized according to the synthetic route described in Scheme S1.[5] 4,5-Dimethoxy-2-nitrobenzaldehyde (1) (2.11 g, 10 mmol) was dissolved in anhydrous THF (50 mL), and cooled to 0 ^{o}C. $NaBH_4$ (380 mg, 10 mmol) was added to the solution. The mixture was stirred for 6 h. The reaction was quenched by addition of water (100 mL) and extracted with CH_2Cl_2. The organic layer was dried over anhydrous Na_2SO_4, followed by filtration, evaporation of the volatiles to give 4,5-Dimethoxy-2-nitrobenzyl alcohol as a yellow solid (2, 90% yield, 1.92 g) without further purification.

The 4,5-Dimethoxy-2-nitrobenzyl chloroformate (3) was synthesized via an acylation of the corresponding alcohol (2) (1.92 g, 9 mmol) with triphosgene (2.673 g, 9 mmol) in anhydrous THF in the presence of DIEA (3.5 ml, 20 mmol) for 6 h. The reaction mixture was then washed by water, extracted with CH_2Cl_2. The organic layer was dried over anhydrous Na_2SO_4, and evaporated to generate a yellow solid (3) (quantitative conversion, 2.47 g) ready for the next reaction without further purification.

N-α-Fmoc-L-lysine (Fmoc-Lys-OH, 3.68 g, 10 mmol) was dissolved in anhydrous THF (50 mL). (3) (2.47 g, 9 mmol) dissolved in 50 ml of THF/CH_2Cl_2 (4/1, v/v) and DIEA (3.5 mL, 20 mmol) was added to the solution containing Fmoc-Lys-OH. The reaction was stirred at room temperature (r.t.) for 6 h. The solvents were evaporated and the resulting oil was extracted with EtOAc and H_2O. The organics were dried over anhydrous Na_2SO_4 and evaporated. 4.6 g Fmoc-Lys(DMNB)-OH (**4**, 7.6 mmol, 85% based on (3)) was obtained after purification by flash chromatography. Light was avoided at this step to prevent the decomposition of Fmoc-Lys(DMNB)-OH. ^{1}H NMR (300 MHz, $CDCl_3$) δ 7.73 (d, J = 7.8, 2H), 7.66 (s, 1H), 7.57 (dd, J = 7.4, 2.8, 2H), 7.37 (t, J = 7.6, 2H), 7.28 (t, J = 7.4, 2H), 6.95 (s, 1H), 5.57 (m, 2H), 5.45 (m, 1H), 5.06 (s, 1H), 4.39 (d, J = 6.9, 3H), 4.19 (t, J = 6.8, 1H), 3.92 (s, 6H), 3.22 (m, 2H), 1.92 (m, 1H), 1.79 (m, 1H), 1.56 (m, 2H), 1.44 (m, 2H); ^{13}C NMR (400 MHz, $CDCl_3$) δ 175.9, 156.6, 156.5, 153.6, 148.3, 143.9, 143.7, 141.4, 139.9, 127.9, 127.2, 125.1, 120.1, 110.5, 108.3, 67.3, 64.7, 63.9, 56.5, 53.6, 47.2, 40.7, 31.8, 29.3, 22.3. ESI-MS m/z calcd for $C_{31}H_{33}N_3O_{10}$: 607.2 Da, found ($M+H^+$): 608.3 Da.

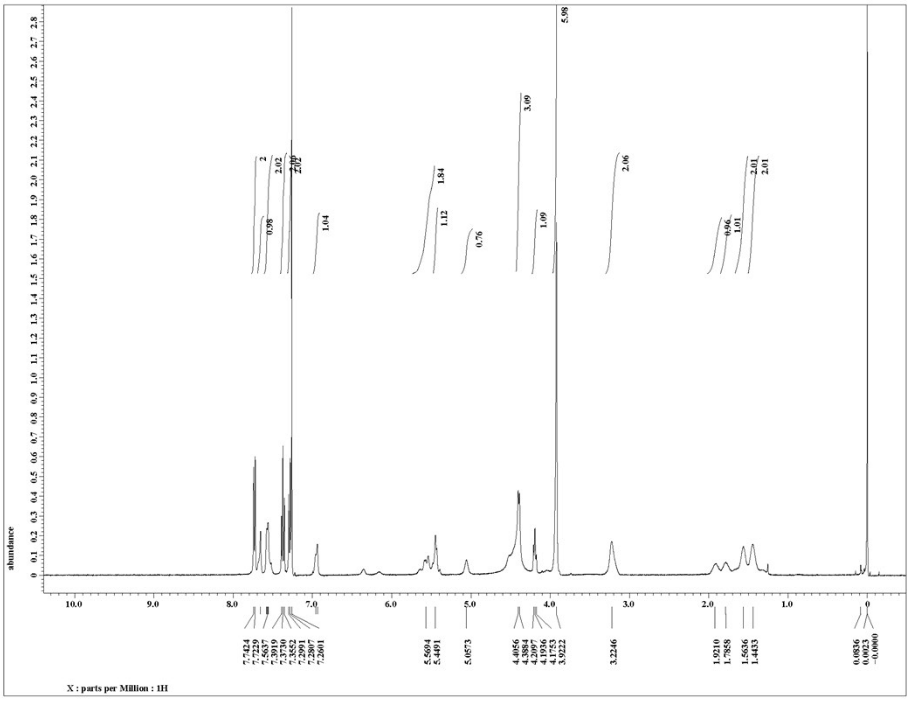

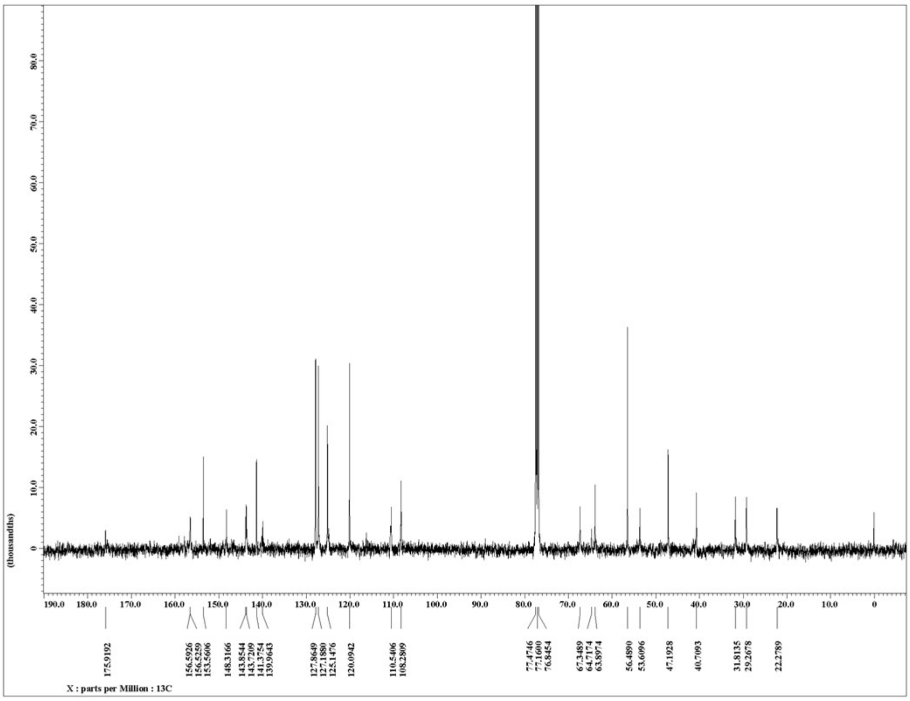

Figure S1. ^{1}H- and ^{13}C-NMR spectra of Fmoc-Lys(DMNB)-OH (**4**).

2. Synthesis of Fmoc-Ser(DMNB)-OH.

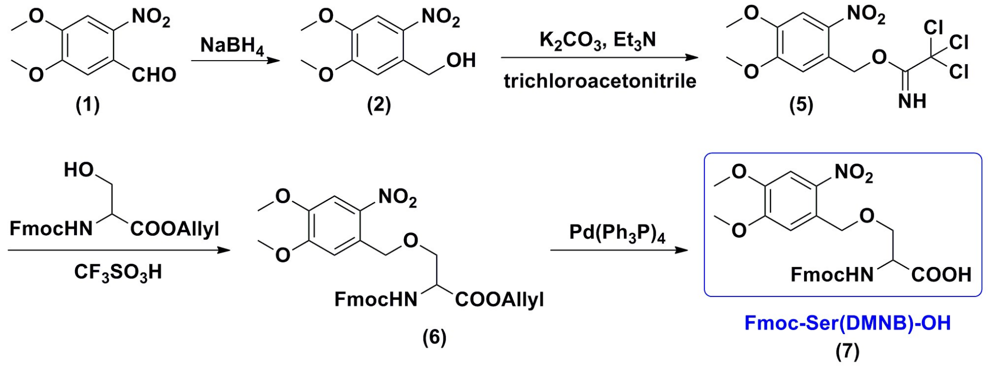

Scheme S2. Synthetic route for Fmoc-Ser(DMNB)-OH.

The synthetic route for Fmoc-Ser(DMNB)-OH was shown in Scheme S2 according to the literature.[6] 4,5-Dimethoxy-2-nitrobenzyl alcohol (2) (1.92 g, 9 mmol) prepared as described above was dissolved in anhydrous CH_2Cl_2 (50 mL) under nitrogen. Anhydrous K_2CO_3 (2.5 g, 18 mmol), trichloroacetonitrile (2.6 g, 18 mmol), and anhydrous triethylamine (1.36 g, 13.5 mmol) were added to the solution and stirred at room temperature for 24 h. CH_2Cl_2 (50 mL) was added to the reaction mixture, and the suspension was washed with 1 M HCl and saturated NaCl. The organic layer was dried over anhydrous Na_2SO_4, and concentrated under reduced pressure to give 4,5-Dimethoxy-2-nitrobenzyl trichloroacetimidate (5) as an orange solid (2.88 g, 8.1 mmol, 90%).

Fmoc-Ser-OAllyl (1.47 g, 4 mmol) and (5) (1.42 g, 4 mmol) was dissolved in anhydrous CH_2Cl_2 (40 mL) under nitrogen. Triflic acid (40 μL) was added to the mixture solution. The resulting dark purple solution was stirred for 20 min at room temperature. Triflic acid (40 μL) was further added twice at 20 min intervals. CH_2Cl_2 (100 mL) and silica gel were directly added to the reaction mixture and then concentrated under reduced pressure, followed by flash chromatography to afford the desired product (6) as a dark brown solid (675 mg, 1.2 mmol, 30%). Light was avoided at the beginning of this step to prevent the decomposition of products.

(6) (675 mg, 1.2 mmol) was dissolved in chloroform (40 mL) and acetic acid (1 mL). N-methylmorpholine (4 mL), and $Pd(PPh_3)_4$ (4.16 g, 3.6 mmol) were added to the solution. The reaction was stirred for 6 h at room temperature, and quenched by addition of 0.5 M HCl (20 mL). The suspension was extracted with EtOAc, and washed with 1 M HCl and saturated NaCl. The organic layer was dried over anhydrous Na_2SO_4, filtered and evaporated under reduced pressure. The residue was purified by flash chromatography to afford Fmoc-Ser(DMNB)-OH (**7**) (550 mg, 1.05 mmol, 88%) as a slight yellow solid. ^{1}H NMR (300 MHz, $CDCl_3$) δ 7.72 (d, J = 7.6, 2H), 7.60 (s, 1H), 7.56 (t, J = 5.8, 2H), 7.38 (t, J = 7.6, 2H), 7.28 (t, J = 7.6, 2H), 7.05 (s, 1H), 5.76 (d, J = 8.3, 1H), 4.93, 4.86 (ABq, J_{AB} = 15.1, 2H), 4.64 (br.s., 1H), 4.50 – 4.36 (m, 2H), 4.19 (t, J = 6.9, 1H), 4.08 (d, J = 9.6, 1H), 3.88 (s, 3H), 3.85 (s, 3H), 3.70 (m, 1H); ^{13}C NMR (300 MHz, $CDCl_3$) δ 174.9, 156.4, 153.8, 147.7, 143.8, 143.6, 141.4, 139.1, 129.6, 127.9, 127.2, 125.1, 120.1, 109.3, 107.9, 70.9, 70.3, 67.4, 56.3, 54.5, 47.1. ESI-MS m/z calcd for $C_{27}H_{26}N_2O_9$: 522.2 Da; found ($M+H^+$): 523.2 Da.

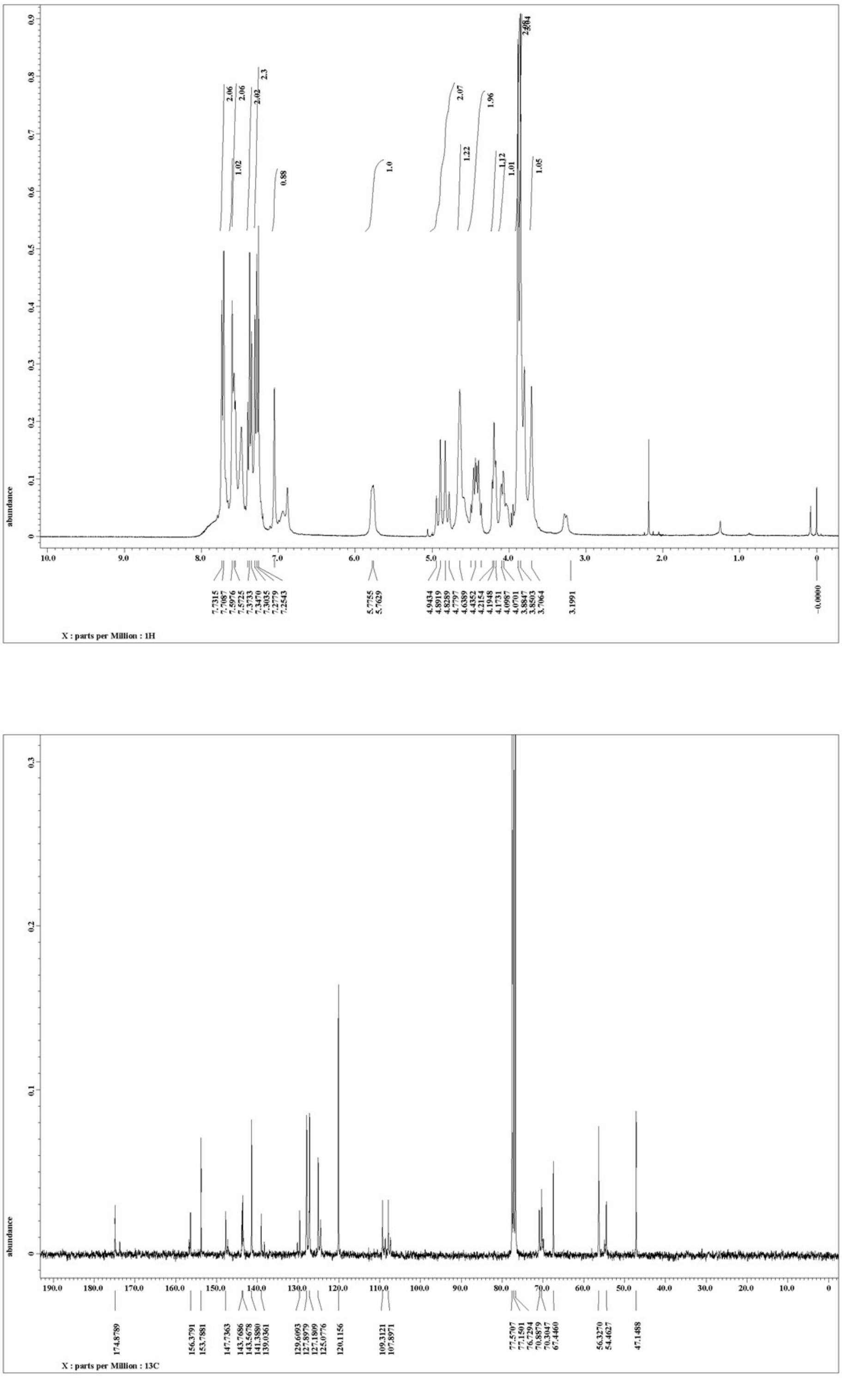

Figure S2. ^{1}H- and ^{13}C-NMR spectra of Fmoc-Ser(DMNB)-OH (**7**).

3. Synthesis of Fmoc-Lys(NPE)-OH.

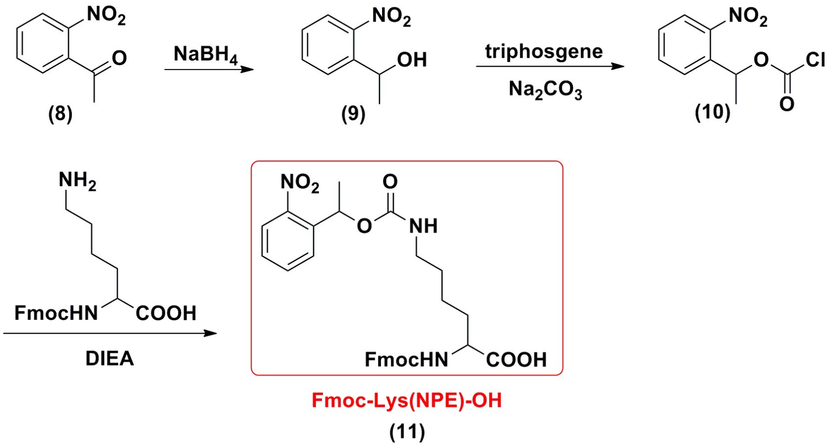

Scheme S3. Synthetic route for Fmoc-Lys(NPE)-OH.

Fmoc-Lys(NPE)-OH was synthesized using the synthetic route described in Scheme S3.[7] Synthesis of 1-(2-nitrophenyl) ethanol (9) was carried out using the same procedure as previously described for (2) which involved the reduction of *o*-Nitroacetophenone (8) (1.65 g, 10 mmol) by $NaBH_4$ (380 mg, 10 mmol) in anhydrous THF (50 mL). 1.5 g (9) (9 mmol, 90%) was obtained without purification.

The 1-(2-nitrophenyl) chloroformate (10) was synthesized via an acylation of the corresponding alcohol (2) with triphosgene (2.67 g, 9 mmol) in anhydrous THF in the presence of Na_2CO_3 (1.06 g, 10 mmol) for 12 h. The presence Na_2CO_3 prevented dehydration of (9) to the corresponding styrene. The reaction mixture was then washed by water, extracted with CH_2Cl_2. The organic layer was dried over anhydrous Na_2SO_4, and evaporated to afford a yellow solid (10) (quantitative conversion, 2.0 g).

N-α-Fmoc-L-lysine (Fmoc-Lys-OH, 3.3 g, 9 mmol) was dissolved in anhydrous THF (50 mL). (10) (2.06 g, 9 mmol) dissolved in 50 ml of THF/DCM (4/1, v/v) and DIEA (3.5 mL, 20 mmol) were added to the solution containing Fmoc-Lys-OH. The reaction was stirred at room temperature for 12 h. The solvents were evaporated and the resulting oil was extracted with EtOAc and H_2O. The organic layer was dried over anhydrous Na_2SO_4 and concentrated under reduced pressure. 4.0 g Fmoc-Lys(NPE)-OH (11, 7.2 mmol, 80% based on (3)) was obtained after purification by flash chromatography. Light was avoided at this step to prevent the decomposition of Fmoc-Lys(NPE)-OH. ^1H NMR (300 MHz, $CDCl_3$) δ 7.89 (d, J = 7.8, 1H), 7.75 (d, J = 6.9, 2H), 7.58 (m, 4H), 7.38 (t, J = 6.9, 3H), 7.29 (t, J = 6.8, 3H), 6.22 (dd, J = 11.9, 6.0, 1H), 5.50 (t, J = 8.0, 1H), 4.89 (s, 1H), 4.40 (m, 3H), 4.20 (m, 1H), 3.12 (m, 2H), 1.85 (m, 1H), 1.66 (m, 1H), 1.59 (dd, J = 9.6, 5.5, 2H), 1.50 (m, 2H), 1.38 (m, 2H); ^{13}C NMR (300 MHz, $CDCl_3$) δ 176.7, 156.3, 155.6, 147.6, 143.8, 143.7, 141.3, 138.5, 133.5, 128.2, 127.7, 127.1, 125.1, 124.4, 120.0, 68.8, 67.2, 53.5, 47.1, 40.5, 31.7, 29.2, 21.2, 21.1. ESI-MS m/z calcd for $C_{30}H_{31}N_3O_8$: 561.2 Da; found ($M+H^+$): 562.1 Da.

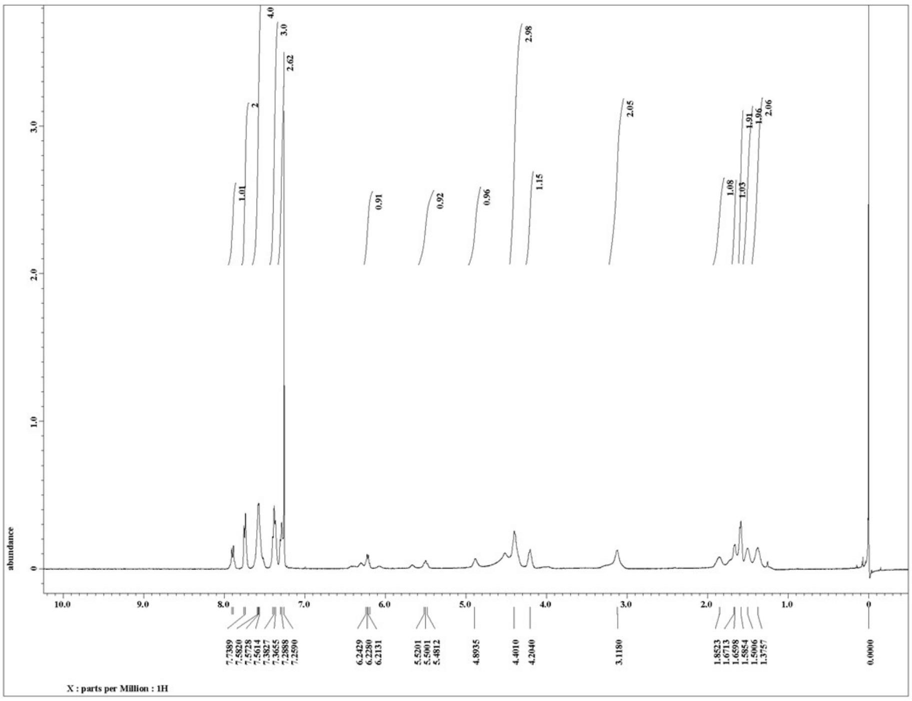

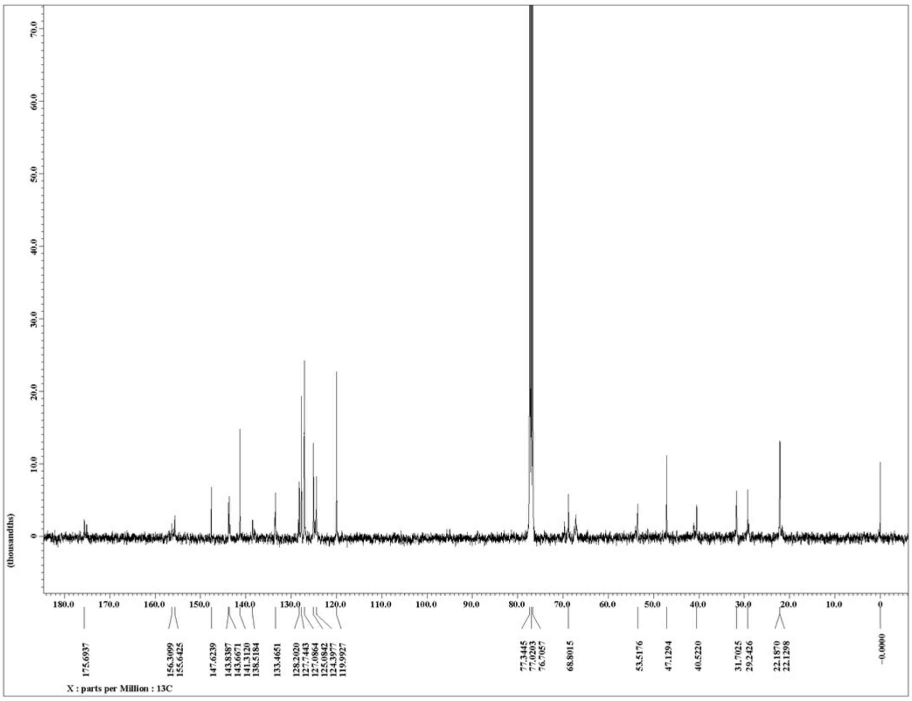

Figure S3. ^{1}H- and ^{13}C-NMR spectra of Fmoc-Lys(NPE)-OH (**11**).

4. Synthesis of Fmoc-Asp(MNI)-OH (12).

Fmoc-Asp(MNI)-OH was synthesized according to the literature.[8] Fmoc-Asp(MNI)-OH. **^{1}H-NMR** (300 MHz, $CDCl_3$): δ 9.04 (br, 1H), 7.80 (s, 1H), 7.75 (d, *J* = 7.6, 2H), 7.63 (t, *J* = 6.9, 2H), 7.38 (t, *J* = 7.6, 2H), 7.32 (dd, *J* = 7.6, 1.4, 1H) 7.30 (dd, *J* = 7.9, 1.4, 1H), 6.68 (d, *J* = 9.3, 1H), 6.19 (d, *J* = 7.6, 1H), 4.30 (m, 4H), 4.24 (t, *J* = 7.2, 2H), 3.91 (s, 3H), 3.36 (dd, *J* = 16.8, 2.7, 1H), 3.10 (q, *J* = 7.9, 2H), 3.02 (dd, *J* = 16.8, 5.1, 1H).

^{13}C-NMR (300 MHz, $CDCl_3$): 172.32, 168.17, 158.41, 155.65, 143.42, 140.55, 135.77, 134.60, 127.25, 126.70, 124.90, 124.69, 122.64, 119.48, 106.33, 65.98, 55.65, 50.23, 49.46, 46.54, 36.78, 25.76. ESI-MS m/z calcd for $C_{28}H_{25}N_3O_8$: 531.2 Da; found $(M\text{-}H)^-$ 530.4 Da.

5. High resolution mass of the photocaged amino acids. $[M\text{-}H]^-$ was measured, and the results were listed in Table S1.

Table S1. High resolution mass of the photocaged amino acids.

Molecule	Fmoc-Lys(NPE)-OH	Fmoc-Lys(DMNB)-OH	Fmoc-Ser(DMNB)-OH	Fmoc-Asp(MNI)-OH
Calc. Mass	560.2033	606.2088	521.1560	530.1563
Observ. Mass	560.2042	606.2084	521.1567	530.1555
mDa	0.9	-0.4	0.7	-0.5
PPM	1.6	-0.7	1.3	-1.5
DBE	17.5	17.5	16.5	18.5
i-FIT	668.1	777.3	618.6	656.2
Norm	0	0.076	0.021	0.003
Conf(%)	99.98	92.71	97.91	99.67
Formula	$C_{30}H_{30}N_3O_8$	$C_{31}H_{32}N_3O_{10}$	$C_{27}H_{25}N_2O_9$	$C_{28}H_{24}N_3O_8$

Part II: Synthesis of HEL and its derivatives

All the peptides derived from HEL and four photocaged HEL variants were shown in Figure S4.

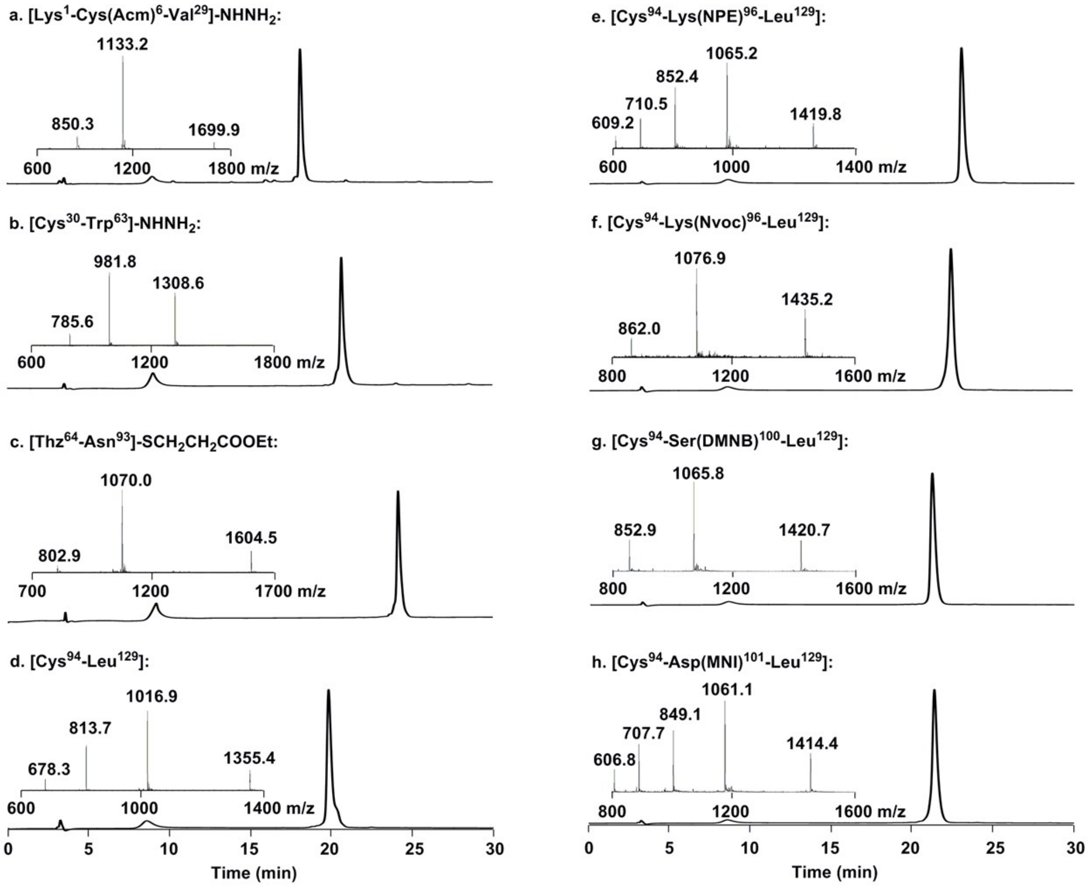

Figure S4. Analytical HPLC (λ = 214 nm) and mass data derived from HEL and four photocaged HEL variants. Each calculated mass is the high point of isotope envelope. (a) [Lys^{1}-Cys(Acm)^{6}-Val^{29}]-$NHNH_2$, calcd: 3396.7 Da, found: 3396.8 Da. (b) [Cys^{30}-Trp^{63}]-$NHNH_2$， calcd: 3922.8 Da, found: 3923.0 Da. (c) [Thz^{64}-Asn^{93}]-SCH_2CH_2COOEt, calcd: 3206.5 Da, found: 3207.2 Da. (d) [Cys^{94}-Leu^{129}], calcd: 4063.0 Da, found: 4063.5 Da. (e) [Cys^{94}-Lys(NPE)^{96}-Leu^{129}], calcd: 4257.1 Da, found: 4257.0 Da. (f) [Cys^{94}-Lys(Nvoc)^{96}-Leu^{129}], calcd: 4303.1 Da, found: 4303.7 Da. (g) [Cys^{94}-Ser(DMNB)^{100}-Leu^{129}], calcd: 4259.1 Da, found: 4259.3 Da. (h) [Cys^{94}-Asp(MNI)^{101}-Leu^{129}], calcd: 4240.1 Da, found: 4240.4 Da. Chromatographic separations were performed by using a linear gradient (20-50%) of buffer B in buffer A over 30 min after an initial isocratic phase of 1% buffer B in buffer A for 2 min on a C4 column with flow rate of 1 mL/min.

HEL and four photocaged HEL variants were synthesized using the same synthetic route described in Figure 1A. Here, taken the convergent chemical synthesis of HEL-K_{96}NPE as an example, the detailed procedure was as follows:

1. Assembly of the N-terminal half [Lys^{1}-Trp^{63}]-$NHNH_2$. [Lys^{1}-Cys(Acm)^{6}-Val^{29}]-$NHNH_2$ (6.8 mg, 2 μmol) dissolved in 200 μL 0.2 M phosphate solution containing 6 M Gn·HCl at pH 3.0, was oxidized by $NaNO_2$ (0.5 M aqueous solution, 20 μmol) in a -15 °C ice-salt bath for 15 min.

[Cys^{30}-Trp^{63}]-NHNH$_2$ (8 mg, 2 μmol) and MPAA (10.1 mg, 60μmol) dissolved in 100 μL 0.2 M phosphate solution containing 6 M Gn·HCl (pH 6.0) were added to the reaction mixture, followed by pH adjustment to 6.5 with 6 M NaOH. The reaction was proceeded smoothly for 8 h and quenched by adding 500 μL 0.1 M TCEP solution containing 6 M Gn·HCl at pH 6.0. The ligation product [Lys^1-Cys(Acm)^6-Trp^{63}]-NHNH$_2$ was purified and lyophilized (8.2 mg, 56% isolated yield). To [Lys^1-Cys(Acm)^6-Trp^{63}]-NHNH$_2$ (8.2 mg, 1.12 μmol) dissolved in 2 mL 50% (vol/vol) acetonitrile-water containing 0.1% (vol/vol) TFA, AgOAc (6.8 mg, 40 μmol) was added to remove the S-Acm side-chain protecting group at Cys^6 overnight. Silver thiolates were converted to free thiols by treatment of a large excess of DTT (62 mg, 400 μmol). A precipitate consisting of Ag-DTT complexes was formed immediately. The precipitate was washed with 50% (vol/vol) acetonitrile-water containing 0.1 M DTT twice, and 0.2 M phosphate solution containing 6 M Gn·HCl (pH 7.2) once. The combined supernatants were purified to afford the N-terminal half [Lys^1-Trp^{63}]-NHNH$_2$ (3.3 mg, 0.46 μmol, 40% isolated yield). The overall yield was 22%. The whole process was monitored by analytical HPLC and ESI-MS (Figure S5).

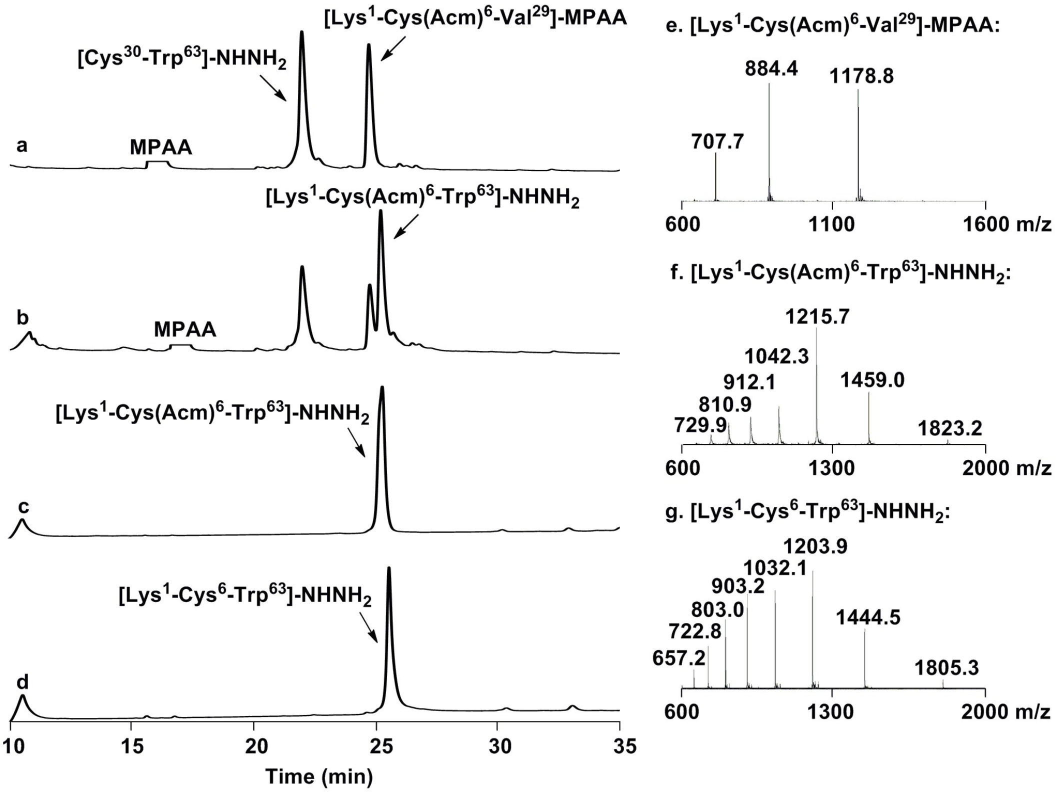

Figure S5. Assembly of the N-terminal half ([Lys^1-Trp^{63}]-NHNH$_2$) of HEL. Analytic HPLC traces for Reaction mixture at (a) t = 5 min, (b) t = 3 h, and (c) Purified [Lys^1-Cys(Acm)^6-Trp^{63}]-NHNH$_2$ after ligation of 8 h. (d) Purified [Lys^1-Cys^6-Trp^{63}]-NHNH$_2$ after removal of S-Acm side-chain protecting group by AgOAc/DTT strategy. ESI-MS of (e) [Lys^1-Cys(Acm)^6-Val^{29}]-MPAA, cacld: 3533.7 Da, found: 3533.5 Da. (f) [Lys^1-Cys(Acm)^6-Trp^{63}]-NHNH$_2$, cacld: 7288.5 Da, found: 7288.9 Da. (g) [Lys^1-Cys^6-Trp^{63}]-NHNH$_2$, calcd: 7217.5 Da, found: 7217.7 Da. Chromatographic separations were performed by using a linear gradient (20-60%) of buffer B in buffer A over 40 min after an initial isocratic phase of 1% buffer B in buffer A for 2 min on a C4 column with flow rate of 1 mL/min.

2. Assembly of the C-terminal half [Cys^{64}-Lys(NPE)^{96}-Leu^{129}]. Ligation of [Thz^{64}-Asn^{93}]-

SCH_2CH_2COOEt (3.2 mg, 1 μmol) and [Cys[94]-Lys(NPE)[96]-Leu[129]] (4.3 mg, 1 μmol) was carried out in 500 μL 0.2 M phosphate solution containing 6 M Gn·HCl, 0.2 M MPAA and 0.1 M TCEP, pH 6.5. The reaction was completed within 2 h. Conversion of Thz to Cys was achieved readily by treatment with $MeONH_2$·HCl (21 mg, 250 μmol, dissolved in 500 μL 0.2 M phosphate solution containing 6 M Gn·HCl) at pH 4.0 for another 4 h in the same reaction vessel. These two steps were carried out in a one-pot manner, which was monitored by HPLC and ESI-MS (Figure S6). The resulting C-terminal half [Cys[64]-Lys(NPE)[96]-Leu[129]] was purified in 50% isolated yield (3.7 mg, 0.5 μmol).

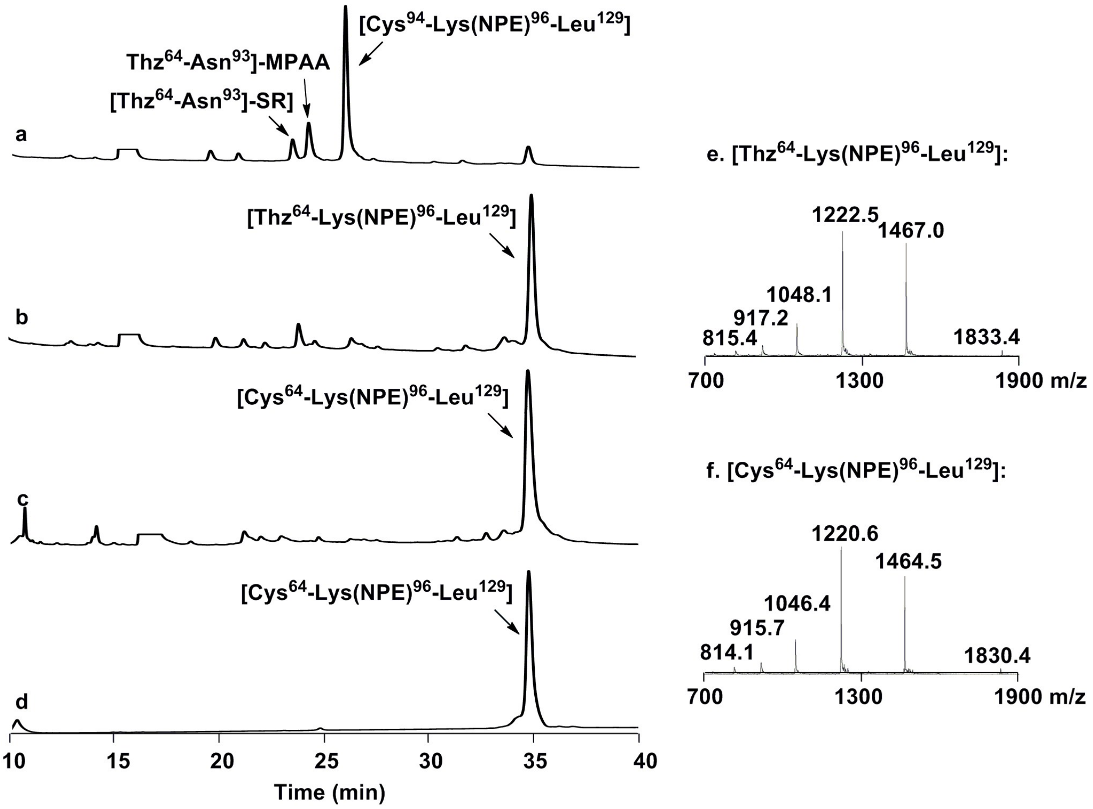

Figure S6. Assembly of the C-terminal half ([Cys[64]-Lys(NPE)[96]-Leu[129]]) of HEL-K_{96}NPE. HPLC analysis (left) and mass determination (right) for (a) reaction about 1 min after mixing of the peptide segments. (b) Reaction mixture after 2 h, followed by (c) $MeONH_2$·HCl treatment for 4 h at pH 4. (d) Purified [Cys[64]-Lys(NPE)[96]-Leu[129]]. ESI-MS of (e) [Thz[64]-Lys(NPE)[96]-Leu[129]], calcd: 7329.5 Da, found: 7329.6 Da. (f) [Cys[64]-Lys(NPE)[96]-Leu[129]], calcd: 7317.5 Da, found : 7317.7 Da. Chromatographic separations were performed as described in the Figure S5 legend.

3. Ligation of N- and C-terminal halves and refolding. The final ligation of [Lys[1]-Trp[63]]-$NHNH_2$ (3.3 mg, 0.46 μmol) with [Cys[64]-Lys(NPE)[96]-Leu[129]] (3.7 mg, 0.5 μmol) was performed smoothly for 8 h under the same conditions described above for the synthesis of [Lys[1]-Cys(Acm)[6]-Trp[63]]-$NHNH_2$. The amount of N-terminal half was slightly more than the C-terminal half. The 129-residue polypeptide chain was obtained in 48% isolated yield (3.5 mg, 0.24 μmol) based on the N-terminal half. The purified full-length [Lys[1]-Lys(NPE)[96]-Leu[129]] was folded by dilution into a refolding buffer containing a redox system (2 mM DTT and 5 mM oxidized glutathione) at pH 8. The final concentration after dilution was around 0.1 mg/mL. After 6 h, the correctly folded HEL-

K_{96}NPE was analyzed and purified by HPLC and characterized by ESI-MS (38% isolated yield, 1.3 mg, 0.09 μmol). The process was monitored by HPLC and ESI-MS, which was shown in Figure S7.

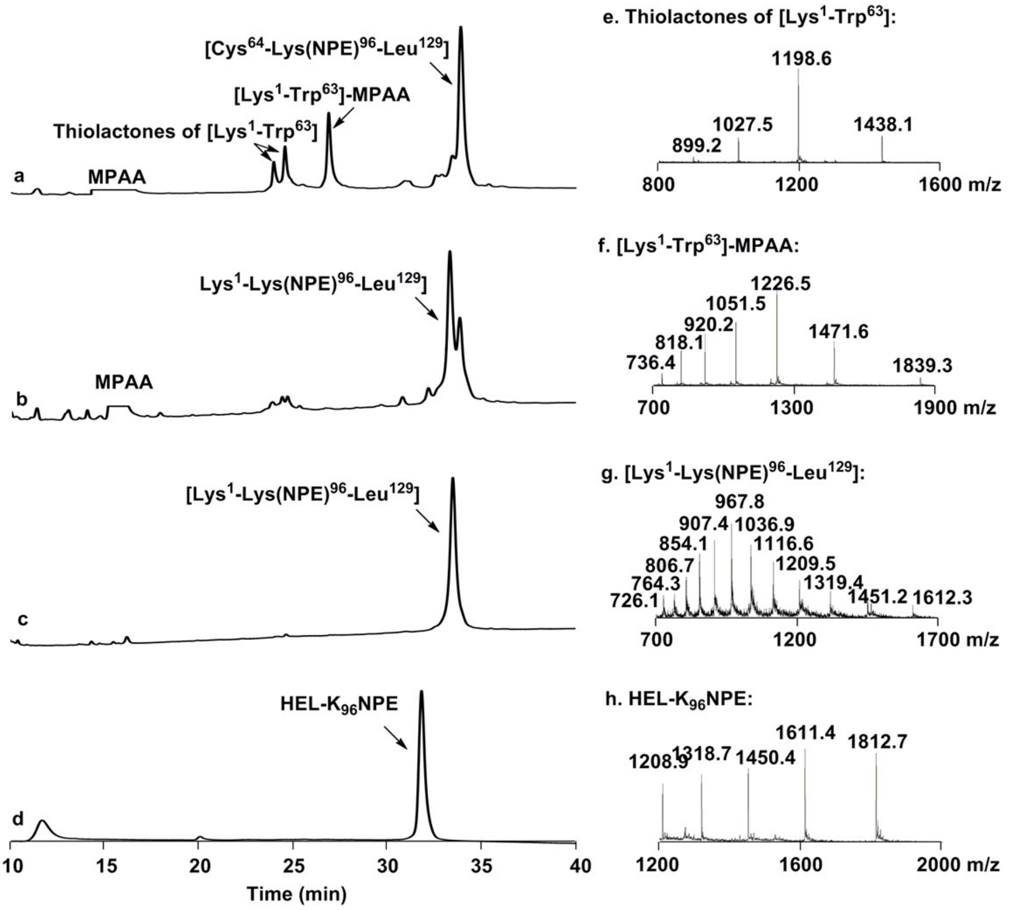

Figure S7. Ligation of the N- and C-terminal halves and refolding of HEL-K_{96}NPE. Analytic HPLC traces for (a) reaction mixture at t = 1 min (Besides the corresponding thioester [Lys^{1}-Trp^{63}]-MPAA, two thiolactones were observed under the ligation conditions owing to the two unprotected internal cysteines inside [Lys^{1}-Trp^{63}]), (b) At t = 2 h. (c) Purified [Lys^{1}-Lys(NPE)^{96}-Leu^{129}]. (d) Purified and folded synthetic HEL-K_{96}NPE after refolding for 6 h in the buffer containing 5 mM oxidized glutathione and 2 mM DTT at pH 8. ESI-MS of (e) thiolactones of [Lys^{1}-Trp^{63}], calcd: 7185.5 Da, found: 7185.6 Da. (f) [Lys^{1}-Trp^{63}]-MPAA, calcd: 7353.5 Da, found: 7353.5 Da. (g) [Lys^{1}-Lys(NPE)^{96}-Leu^{129}], calcd: 14502.0 Da, found: 14502.3 Da. (h) folded HEL-K_{96}NPE, calcd: 14494.0 Da, found: 14494.1 Da. This value corresponded to a loss of 8 Da, which was in excellent agreement with the formation of four disulfides. Chromatographic separations were performed as described in the Figure S5 legend.

4. Synthesis of HEL, HEL-K_{96}Nvoc, HEL-S_{100}DMNB, and HEL-D_{101}MNI. HEL, HEL-K_{96}Nvoc, HEL-S_{100}DMNB, and HEL-D_{101}MNI were synthesized in the same manner. The isolated yield of each HEL variant was listed in Table S2. The isolated yield of HEL was slightly higher than these modified HEL variants. HPLC analysis in a C4 reversed phase chromatography column showed that all four photocaged HEL variants exhibited enhanced hydrophobic features compared to HEL, reflecting hydrophobic character of each photocleavable moiety (Figure S8). The HPLC traces and mass analysis of the intermediates during the synthesis of HEL, HEL-

K_{96}Nvoc, HEL-S_{100}DMNB, and HEL-D_{101}MNI were shown in Figure S9 to S12 respectively.

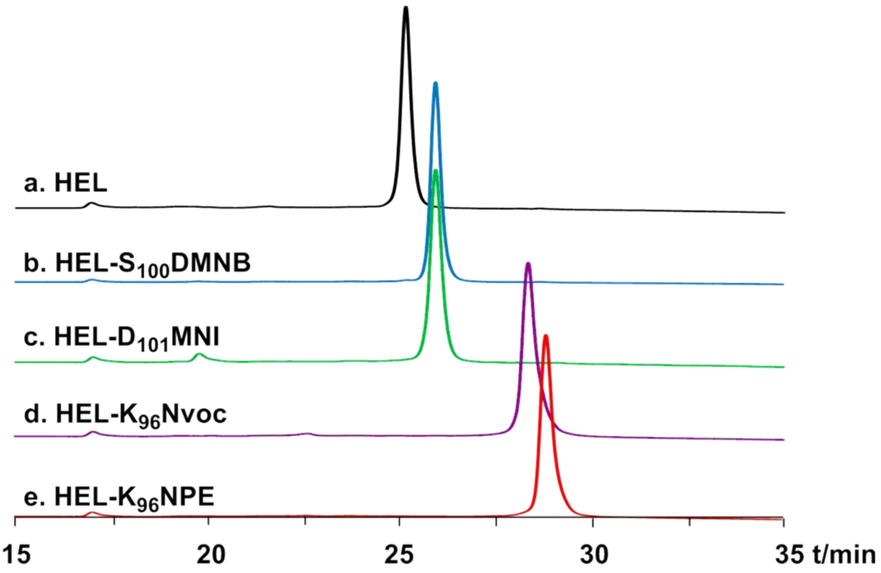

Figure S8. HPLC analysis of folded HEL variants. (a) native HEL, (b) HEL-S_{100}DMNB, (c) HEL-D_{101}MNI, (d) HEL-K_{96}Nvoc, (e) HEL-K_{96}NPE.Chromatographic separations were performed by using a linear gradient (20-50%) of buffer B in buffer A over 30 min after an initial isocratic phase of 1% buffer B in buffer A for 2 min on a C4 column with flow rate of 1 mL/min.

Table S2. Isolated yields of HEL variants.

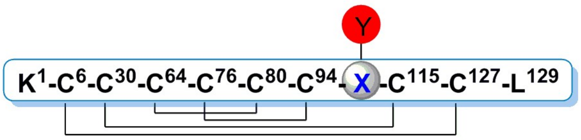

Entry	X	Y	Isolated Yield[a]
HEL	-	-	11%
HEL-K_{96}NPE	Lys96	NPE	9%
HEL-K_{96}Nvoc	Lys96	Nvoc	8%
HEL-S_{100}DMNB	Ser100	DMNB	10%
HEL-D_{101}MNI	Asp101	MNI	10%

[a] The isolated yield is based on the C-terminal peptide.

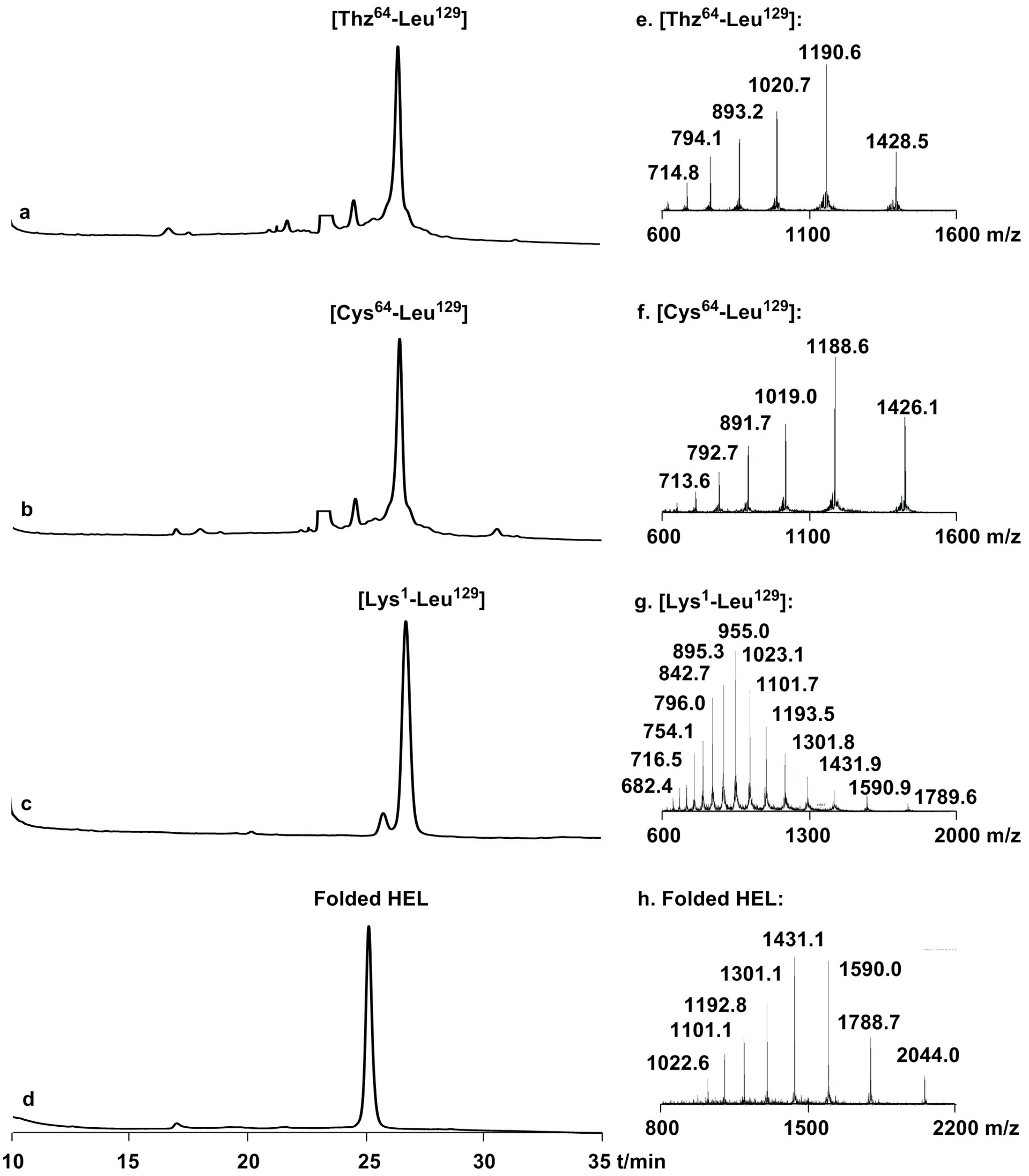

Figure S9. Characterization of the intermediates and final product of HEL. Analytic HPLC traces for (a) ligation of [Thz^{64}-Asn^{93}]-SR and [Cys^{94}-Leu^{129}] for 2 h, followed by (b) $MeONH_2$ treatment at pH 4 for another 4 h. (c) Purified [Lys^{1}-Leu^{129}]. (d) Purified folded native HEL. ESI-MS of (e) [Thz^{64}-Leu^{129}], calcd: 7137.5 Da, found: 7137.7 Da. (f) [Cys^{64}-Leu^{129}], calcd: 7125.5 Da, found: 7125.7 Da. (g) full length [Lys^{1}-Leu^{129}], calcd: 14308.9 Da, found: 14309.2 Da. (h) folded HEL, calcd: 14300.9 Da, found: 14301.7 Da. This value corresponded to a loss of 8 Da, which was in excellent agreement with the formation of four disulfides. Chromatographic separations were performed as described in the Figure S5 legend.

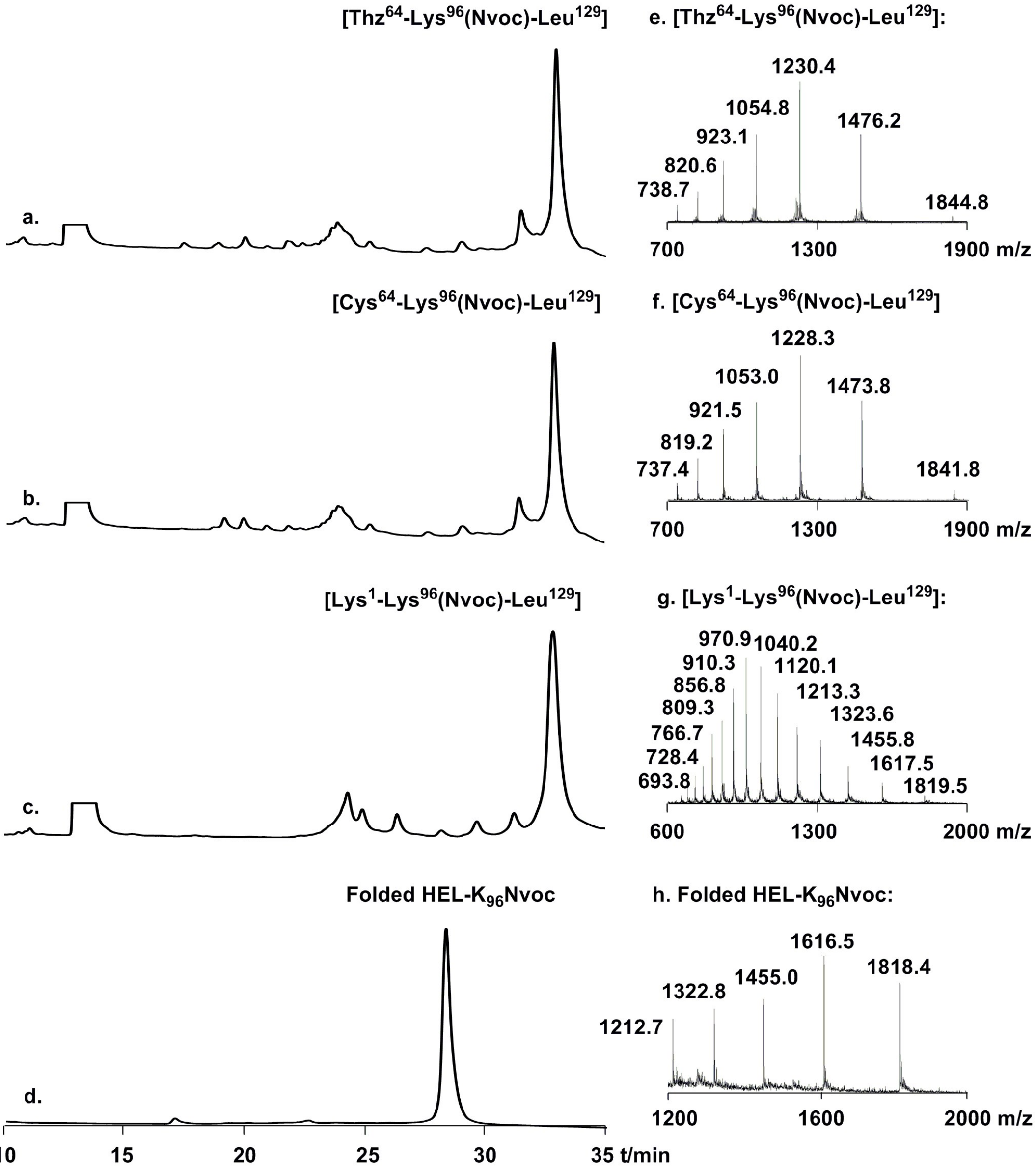

Figure S10. Characterization of the intermediates and final product of HEL-K$_{96}$Nvoc. Analytic HPLC traces for (a) ligation of [Thz^{64}-Asn^{93}]-SR and [Cys^{94}-K^{96}(Nvoc)-Leu^{129}] for 2 h, followed by (b) MeONH$_2$ treatment at pH 4 for another 4 h. (c) Ligation of [Lys^{1}-Trp^{63}]-NHNH$_2$ and [Cys^{64}-K^{96}(Nvoc)-Leu^{129}] for 6 h produced [Lys^{1}-K^{96}(Nvoc)-Leu^{129}]. (d) Purified folded HEL-K$_{96}$Nvoc. ESI-MS of (e) [Thz^{64}-K^{96}(Nvoc)-Leu^{129}], calcd: 7375.5 Da, found: 7376.3 Da. (f) [Cys^{64}-K^{96}(Nvoc)-Leu^{129}], calcd: 7363.5 Da, found: 7363.8 Da. (g) full length [Lys^{1}-K^{96}(Nvoc)-Leu^{129}], calcd: 14548.0 Da, found: 14548.5 Da. (h) folded HEL, calcd: 14540.0 Da, found: 14540.0 Da. This value corresponded to a loss of 8 Da, which was in excellent agreement with the formation of four disulfides. Chromatographic separations were performed as described in the Figure S5 legend.

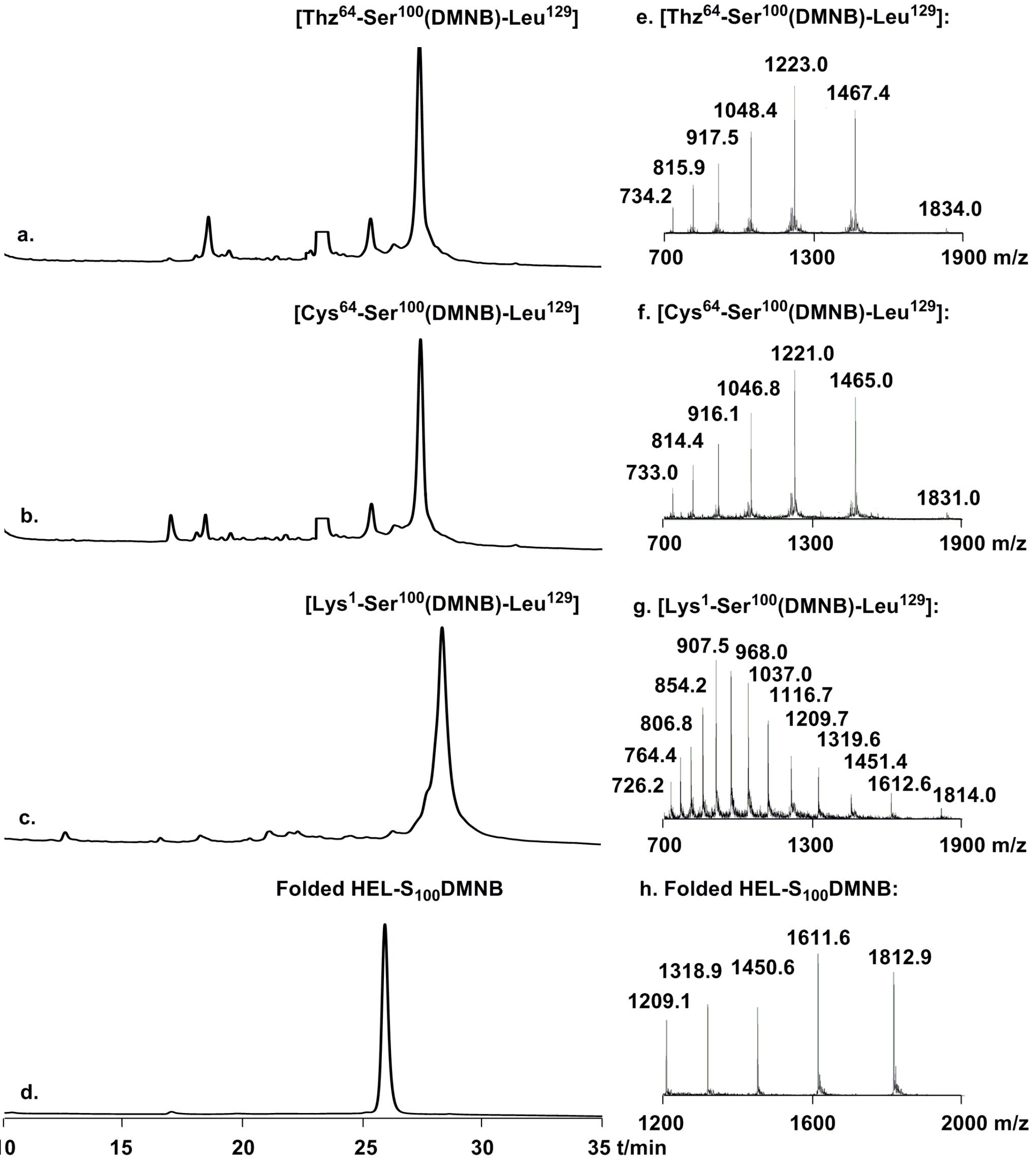

Figure S11. Characterization of the intermediates and final product of HEL-S$_{100}$DMNB. Analytic HPLC traces for (a) ligation of [Thz^{64}-Asn^{93}]-SR and [Cys^{94}-S^{100}(DMNB)-Leu^{129}] for 2 h, followed by (b) $MeONH_2$ treatment at pH 4 for another 4 h. (c) [Lys^{1}-S^{100}(DMNB)-Leu^{129}]. (d) Purified folded HEL-S$_{100}$DMNB. ESI-MS of (e) [Thz^{64}-S^{100}(DMNB)-Leu^{129}], calcd: 7331.5 Da, found: 7332.0 Da. (f) [Cys^{64}-S^{100}(DMNB)-Leu^{129}], calcd: 7319.5 Da, found: 7320.3 Da. (g) full length [Lys^{1}-S^{100}(DMNB)-Leu^{129}], calcd: 14504.0 Da, found: 14504.3 Da. (h) folded HEL-S$_{100}$DMNB, calcd: 14496.0 Da, found: 14496.1 Da. This value corresponded to a loss of 8 Da, which was in excellent agreement with the formation of four disulfides. Chromatographic separations were performed as described in the Figure S5 legend.

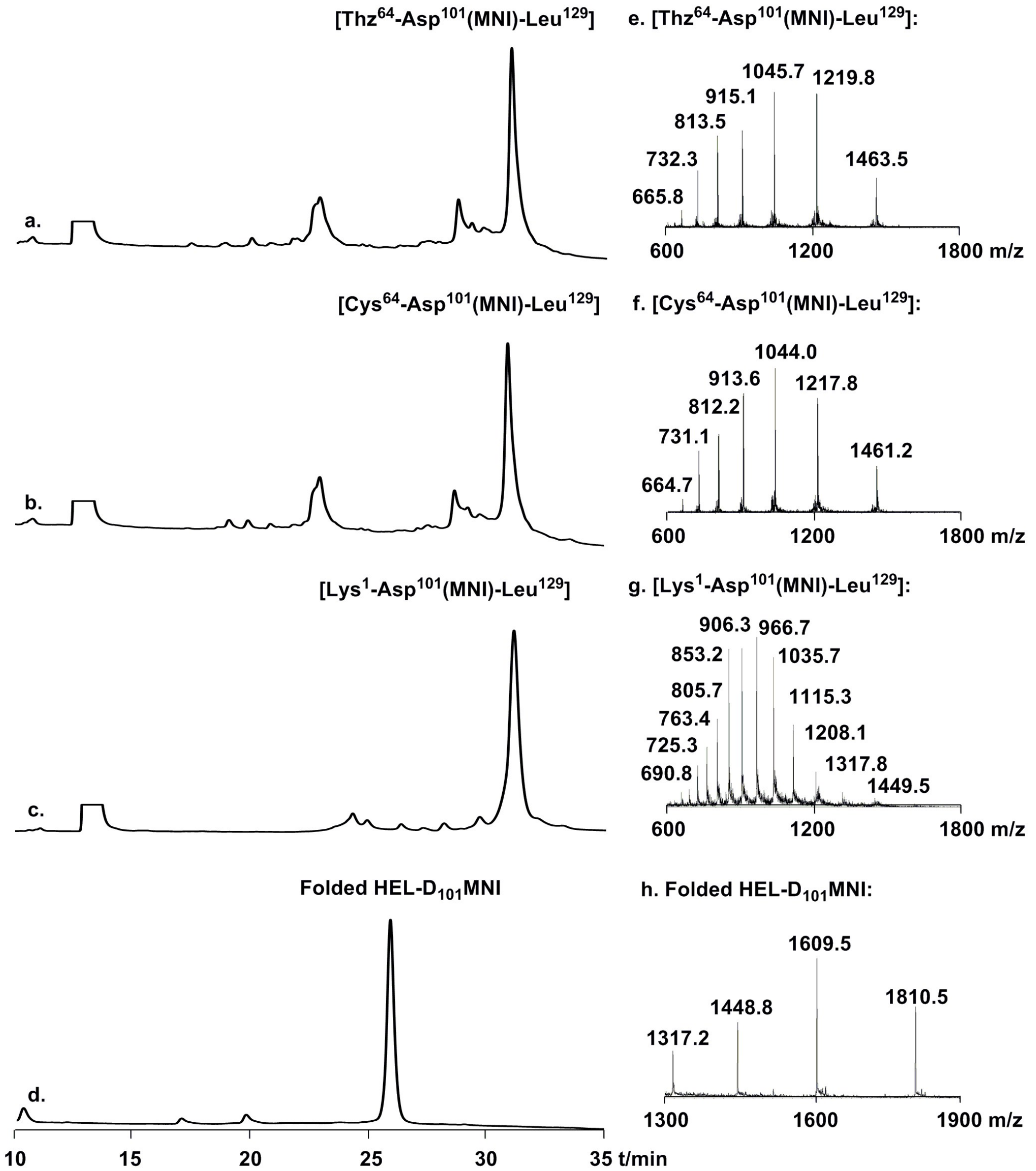

Figure S12. Characterization of the intermediates and final product of HEL-D_{101}MNI. Analytic HPLC traces for (a) ligation of [Thz^{64}-Asn^{93}]-SR and [Cys^{94}-D^{101}(MNI)-Leu^{129}] for 2 h, followed by (b) $MeONH_2$ treatment at pH 4 for another 4 h. (c) Ligation of [Lys^{1}-Trp^{63}]-$NHNH_2$ and [Cys^{64}-D^{101}(MNI)-Leu^{129}] for 6 h produced [Lys^{1}-D^{101}(MNI)-Leu^{129}]. (d) Purified folded HEL-D_{101}MNI. ESI-MS of (e) [Thz^{64}-D^{101}(MNI)-Leu^{129}], calcd: 7312.5 Da, found: 7312.8 Da. (f) [Cys^{64}-D^{101}(MNI)-Leu^{129}], calcd: 7300.5 Da, found: 7300.8 Da. (g) full length [Lys^{1}-D^{101}(MNI)-Leu^{129}], calcd: 14485.0 Da, found: 14485.3 Da. (h) folded HEL-D_{101}MNI, calcd: 14477.0 Da, found: 14477.2 Da. This value corresponded to a loss of 8 Da, which was in excellent agreement with the formation of four disulfides. Chromatographic separations were performed as described in the Figure S5 legend.

5. High Resolution mass data of the synthetic HELs. The full spectra and the ^{13}C isotope distribution of the ion selected as representatives were shown in Figure S13.

1a.

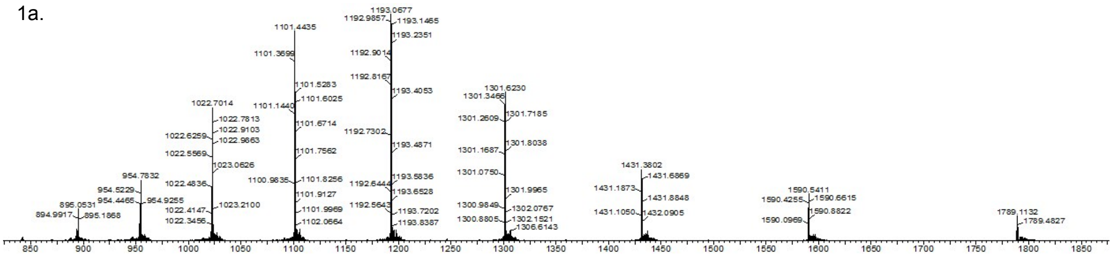

1b. $[M+12H]^{12+}$

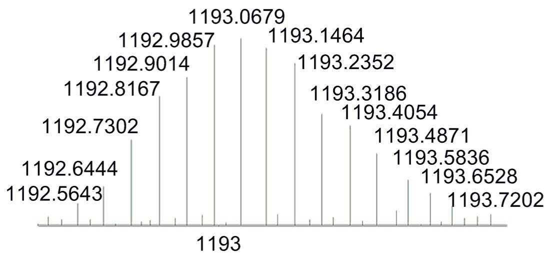

1c. $[M+11H]^{11+}$

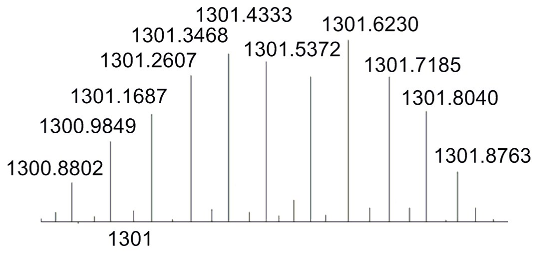

2a.

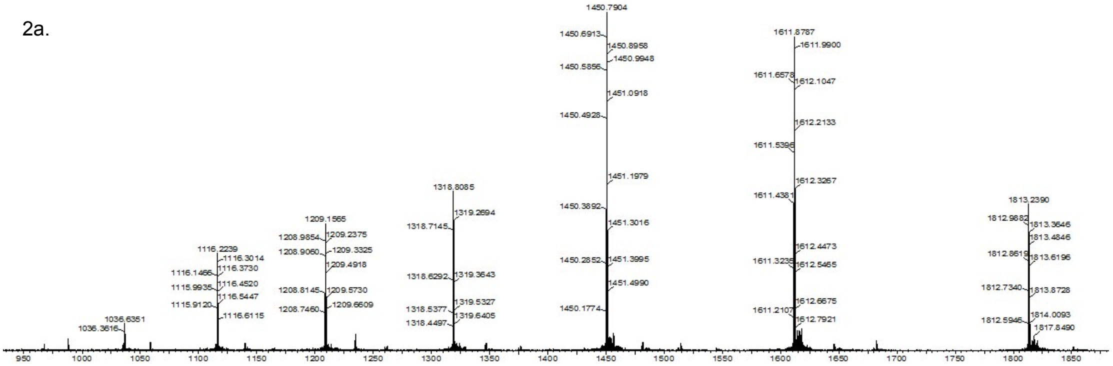

2b. $[M+10H]^{10+}$

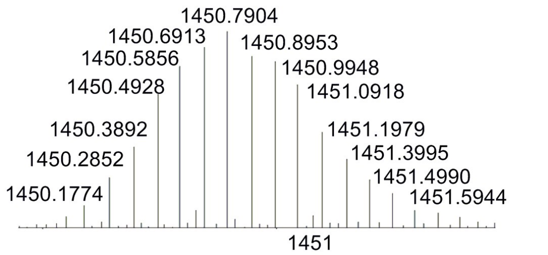

2c. $[M+9H]^{9+}$

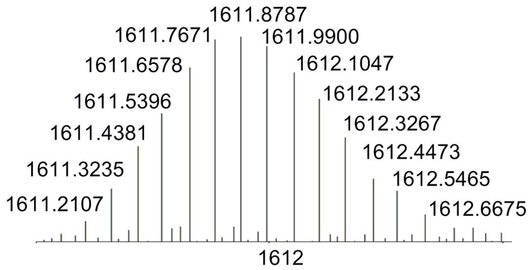

3a.

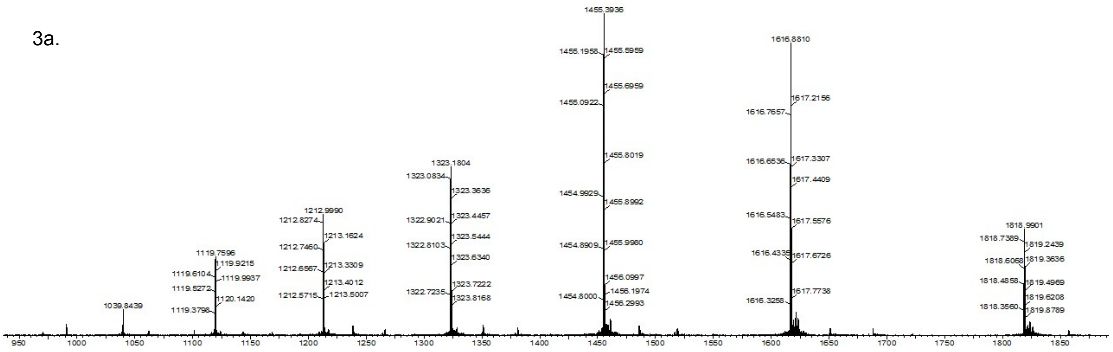

3b. $[M+10H]^{10+}$

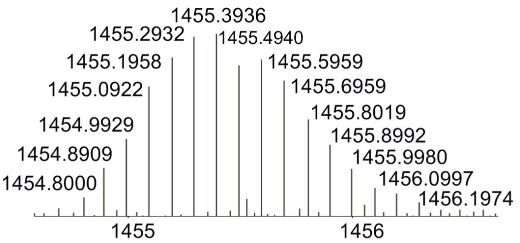

3c. $[M+9H]^{9+}$

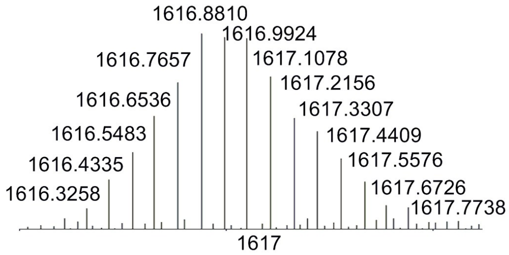

4a.

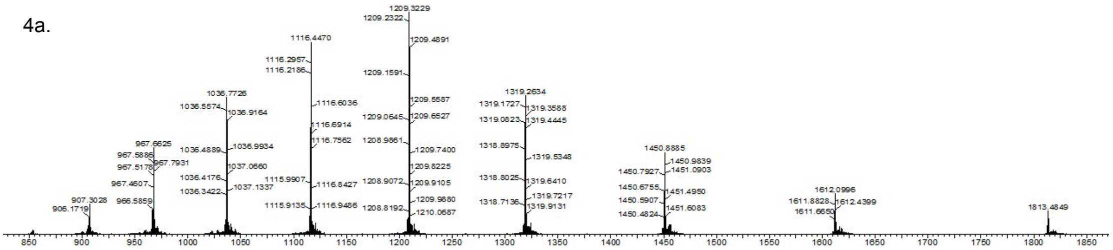

4b. $[M+12H]^{12+}$

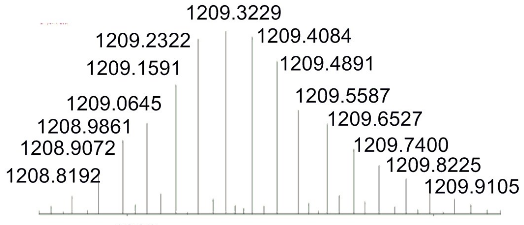

4c. $[M+11H]^{11+}$

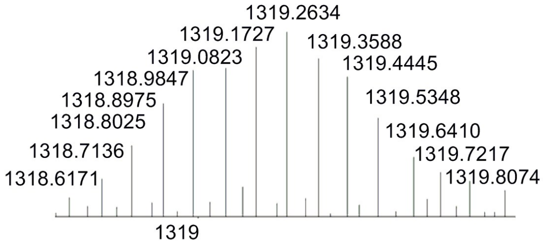

5a.

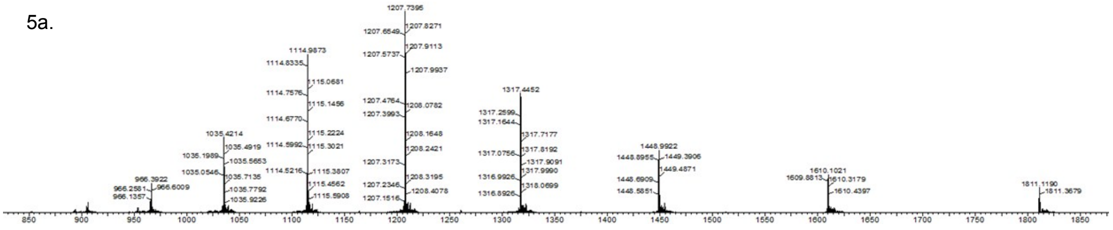

5b. $[M+12H]^{12+}$

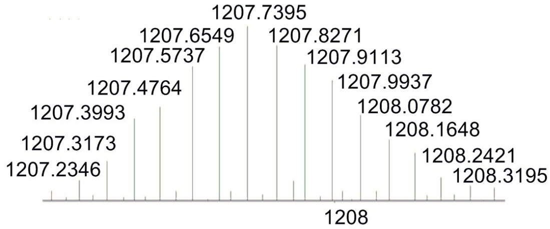

5c. $[M+11H]^{11+}$

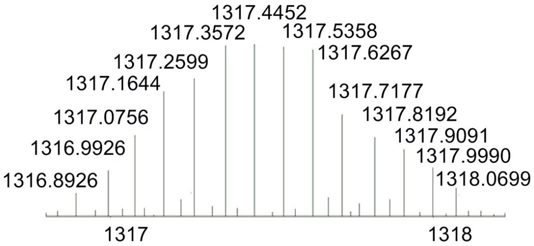

Figure S13. High-resolution mass data of the five synthetic proteins for (1) (a) the mass spectrum of HEL, and the ^{13}C isotope distribution of (1b) $[M+11H]^{11+}$ and (1c) $[M+12H]^{12+}$ as representatives. (2) (a) the mass spectrum of HEL-K_{96}NPE, and the ^{13}C isotope distribution of (2b) $[M+9H]^{9+}$ and (2c) $[M+10H]^{10+}$ as representatives. (3) (a) the mass spectrum of HEL-K_{96}Nvoc, and the ^{13}C isotope distribution of (3b) $[M+9H]^{9+}$ and (3c) $[M+10H]^{10+}$ as representatives. (4) (a) the mass spectrum of HEL-S_{100}DMNB, and the ^{13}C isotope distribution of (4b) $[M+11H]^{11+}$ and (4c) $[M+12H]^{12+}$ as representatives. (5) (a) the mass spectrum of HEL-D_{101}MNI, and the ^{13}C

isotope distribution of (5b) $[M+11H]^{11+}$ and (5c) $[M+12H]^{12+}$ as representatives.

6. (LC-MS)/MS analysis of HEL. Folded, synthetic HEL was treated with DTT, alkylated with iodoacetamide at all Cys residues and digested by trypsin. Eight fragments were observed, which were well matched with the sequence of HEL (Figure S14).

A.

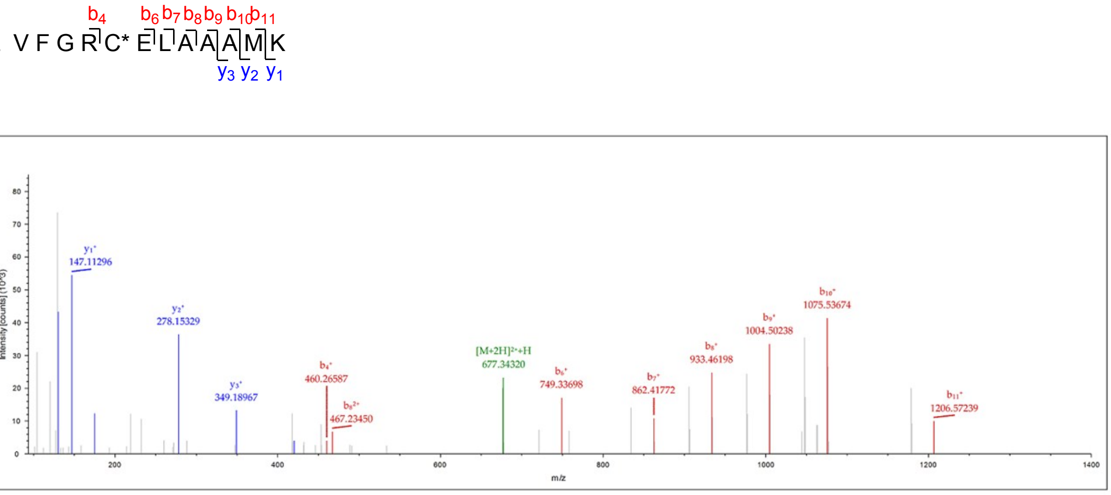

B.

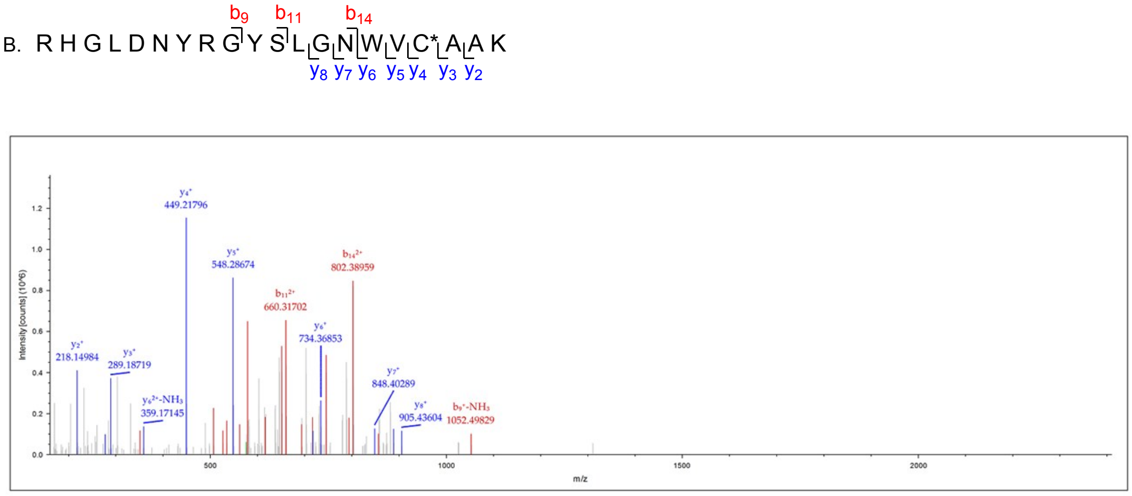

C.

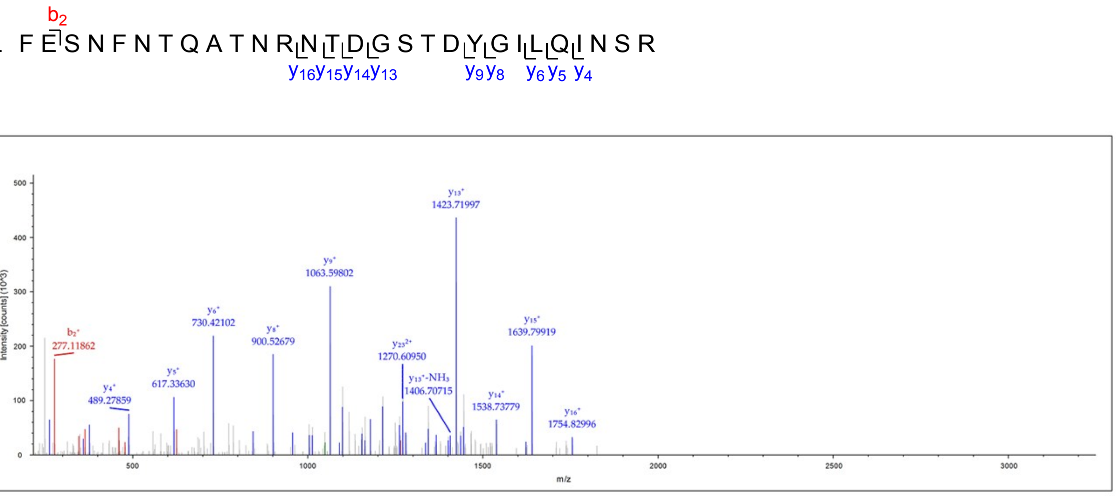

D. WWC*NDGR

y6 y5 y4 y3 y2 y1

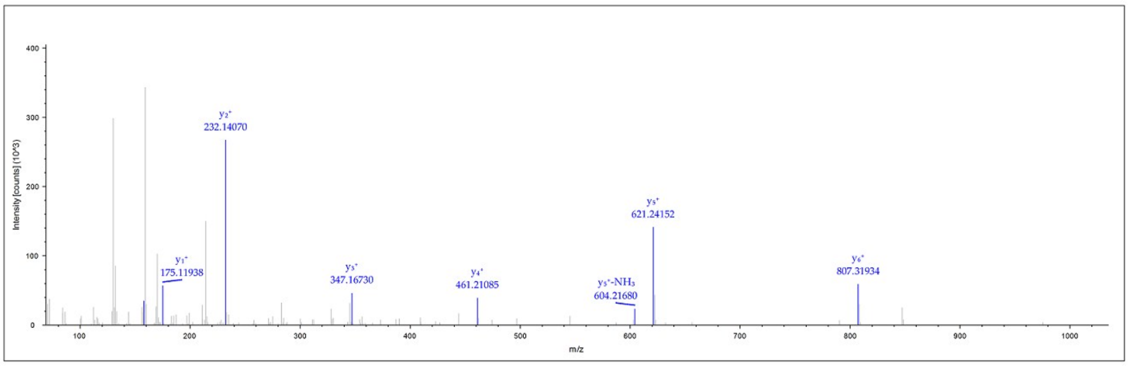

E. T P G S R N L C* N I P C* S A L L S S D I T A S V N C* A K

y13 y12 y9 y8 y7 y6 y5 y4 y3

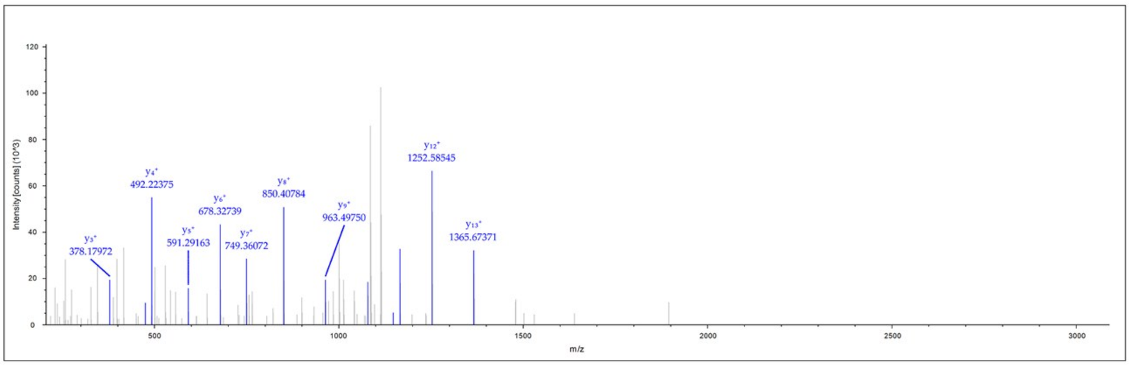

F. K I V S D G N G M N A W V A W R N R

b3

y15 y13 y11 y9 y8 y7 y6 y4 y3 y2 y1

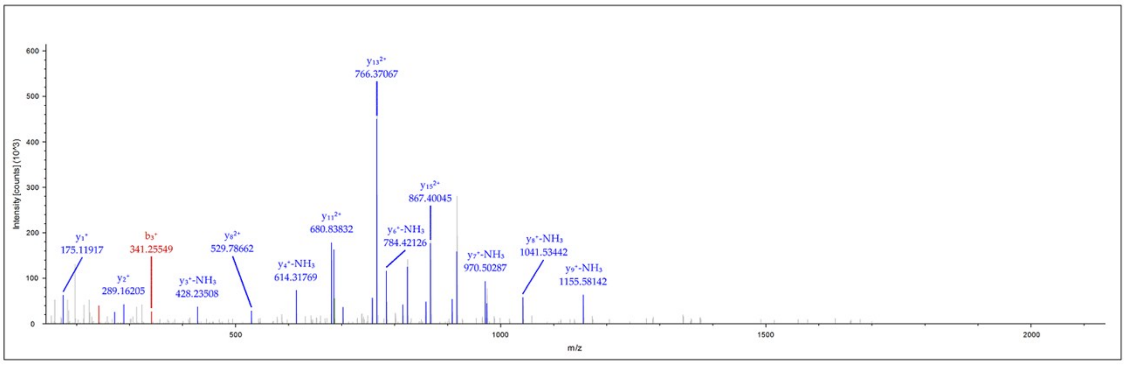

G. C*KGTDVQAWIR

b2

y10 y9 y8 y7 y6 y5 y4 y3 y1

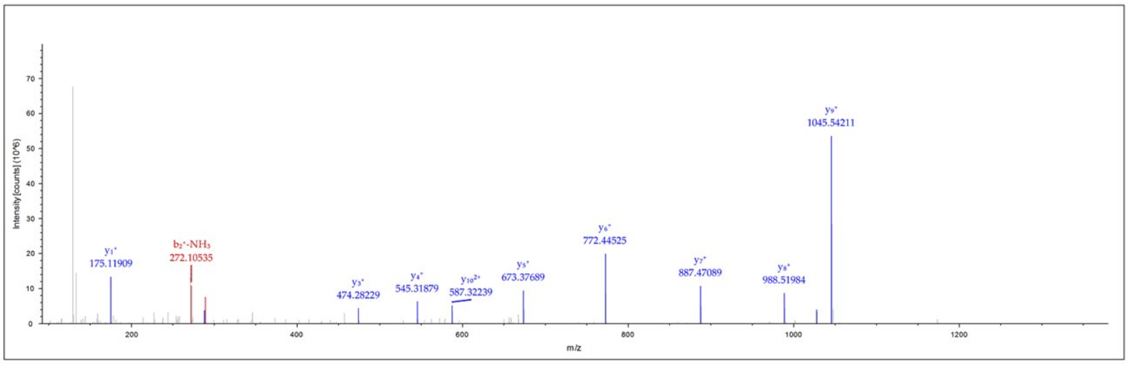

H. 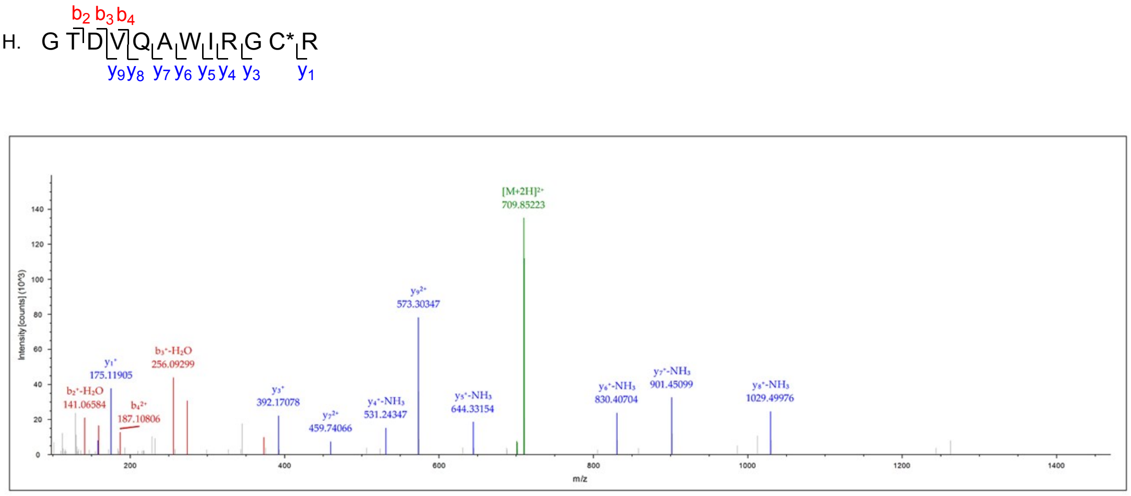

Figure S14. (LC-MS)/MS analysis of synthetic HEL after treatment with DTT and alkylated with iodoacetamide at all Cys residues. The polypeptide (129 aa) was digested by trypsin to eight pieces: (A) VFGRC*ELAAAMK; (B) RHGLDNYRGYSLGNWVC*AAK; (C) FESNFNTQATNRNTDGSTDYGILQINSR; (D) WWC*NDGR; (E) TPGSRNLC*NIPC*SALLSSDITASVNC*AK; (F) KIVSDGNGMNAWVAWRNR; (G) C*KGTDVQAWIR; (H) GTDVQAWIRGCR. Except for residues Lys[1] and Leu[129], which are not detected by LC-MS, all the other amino acid residues are matched with the database. C* represents cysteine alkylated with iodoacetamide.

7. Disulfide bond identification. The disulfide linkages of folded, synthetic HEL was mapped by (LC-MS)/MS analysis (Figure S15). The folded and synthetic HEL was directly digested by trypsin without treatment of DTT. The fragments containing disulfide bond(s) were observed. The linkage between C1 and C8, C2 and C7 were readily identified. However, the sequence containing C3, C4, C5 and C6 (WWCNDGR NLCNIPCSALLSSDITASVNCAK) had only two cleavage sites between C3 and C4, we cannot easily distinguish the disulfide linkages between C3, C4, C5 and C6 just based on the results of trypsin digestion. In the MS/MS results, we did not find peptide segments (b) containing the disulfide bond between C3 and C4, such as WWCNDGR NLC, WWCNDGR NLCN, or WWCNDGR NLCNI, WWCNDGR NLCNIP; we did not find peptide segments (y) containing the disulfide bond between C5 and C6, such as NIPCSALLSSDITASVNCAK, IPCSALLSSDITASVNCAK, PCSALLSSDITASVNCAK, or CSALLSSDITASVNCAK either, indicating that the linkages between C3 and C4, C5 and C6

would not be appropriate. Considering that there were two “D” in the sequence, we used enzyme Asp-n to digest the full length folded HEL without treatment of DTT. Asp-n is prone to cleave the peptide bond at the N-terminus of Asp. However, after careful analysis, we did not observe the peptide fragment containing a disulfide bond of DGRTPGSRNLCNIPCSALLSS, or DITASVNC(WWCN)AK, which contained a disulfide bond between C4 and C5, or C3 and C6. Therefore, we excluded the possibility of disulfide linkages between C3 and C6, C4 and C5. Based on these results, we deduced that the disulfide linkages between these four cysteines should be C3 and C5, C4 and C6.

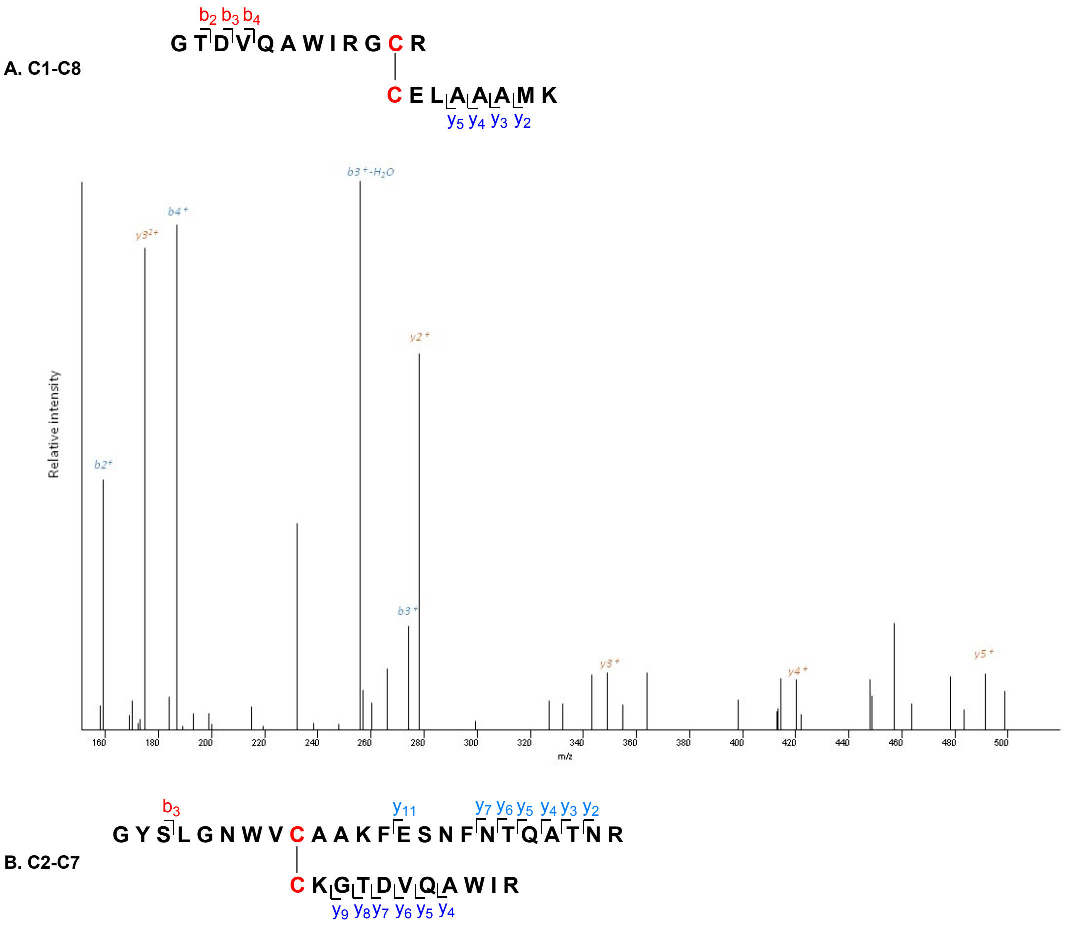

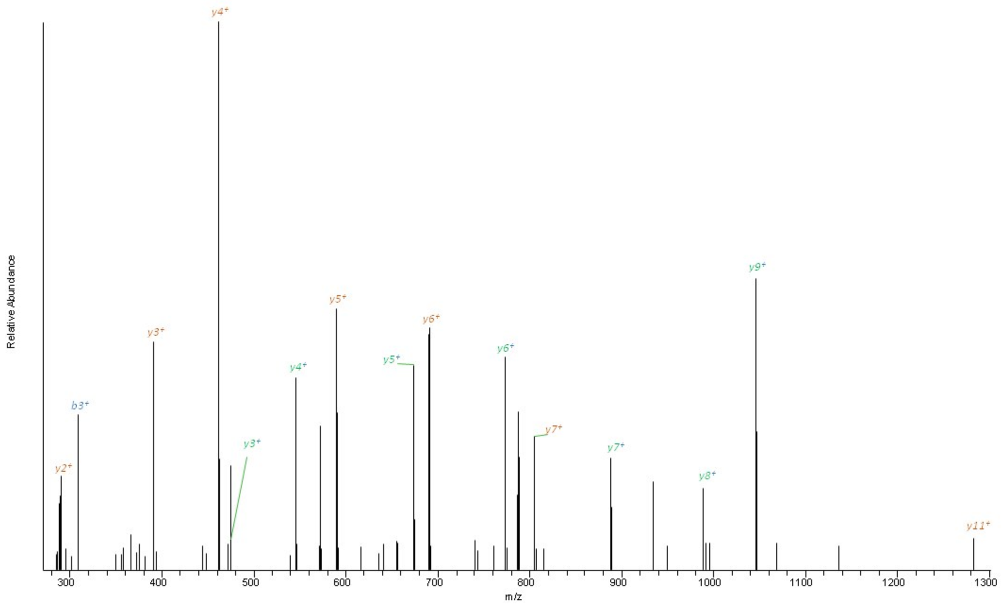

C. C3-C5, C4-C6

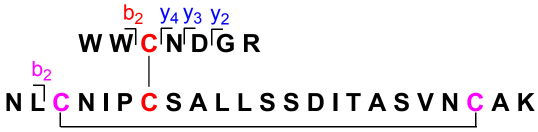

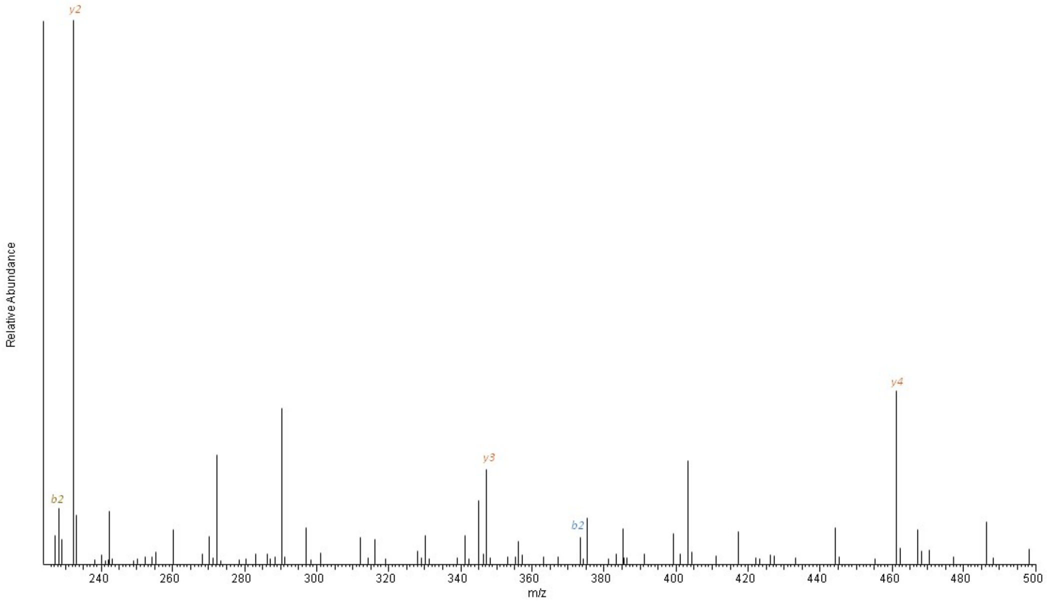

Figure S15. (LC-MS)/MS analysis of synthetic folded HEL directly digested by trypsin. Three polypeptide segments containing disulfide bond linkages were found: (A) the disulfide linkage between C1 and C8; (B) the disulfide linkage between C2 and C7; and (C) the disulfide linkages of C3-C5 and C4-C6.

8. CD spectra of all of the synthetic HELs. CD spectra of synthetic HEL and four caged HEL variants were shown in Figure S16. The final concentration of these proteins was about 10 μM. CD spectra of all the caged proteins exhibited a negative minimum absorption at 208 nm, which was consistent with the HEL purified from hen egg white. These results revealed that attachment of these photosensitive moieties on HEL did not appreciably change the overall structure of HEL.

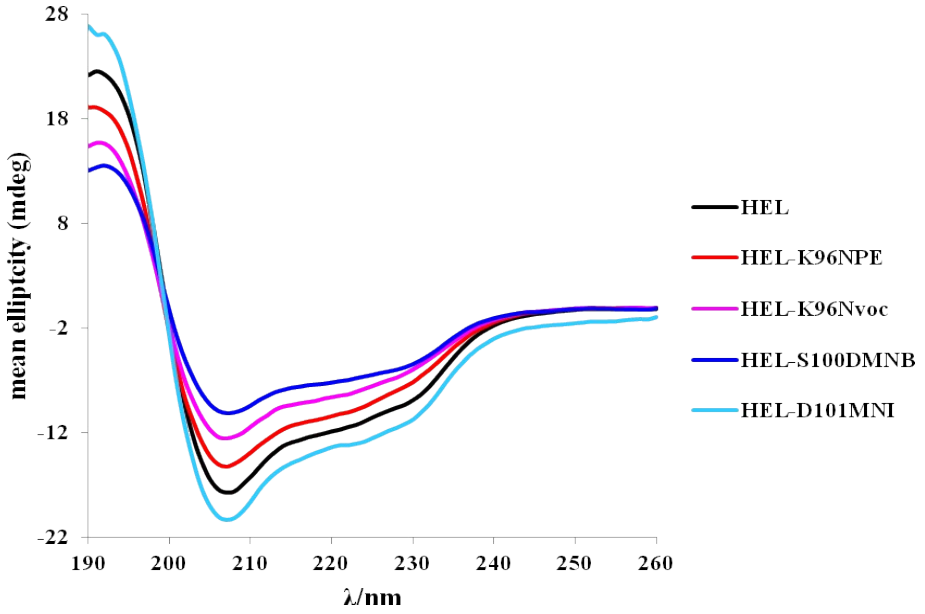

Figure S16. CD spectra of HEL and four photocaged HEL variants.

Part III: Characterization of the antigenicity of HEL variants

1. Binding capacity of synthetic HEL to HyHEL-10 was similar to that of native HEL from hen egg white. Binding capacity of HyHEL-10 antibody to synthetic HEL and native HEL from hen egg white was detected by ELISA. Shown in Figure S17 are binding curves of HyHEL-10 antibody with either type of HEL coated on ELISA plate in a 1:2 serially diluted manner starting from 5 μg/mL. The ELISA data suggested that our synthetic HEL exhibited similar antigenicity to the HyHEL-10 antibody as native HEL from hen egg white.

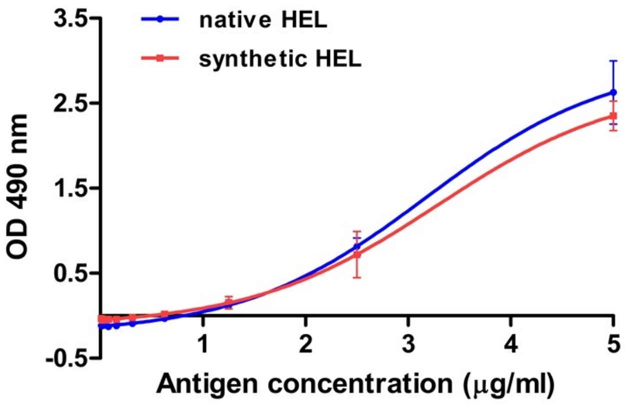

Figure S17. Binding capacity of synthetic HEL and native HEL from hen egg white to HyHEL-10 antibody. Bars represent mean ± SEM.

2. Antigenicity restoration of caged HEL variants after exposure to different time dosage of UV light. Binding capacity of different variants of HEL to HyHEL-10 antibody was tested after irradiation for 30 s or 300 s of UV light (365 nm, 18 mW/cm^2, Figure S18). The results showed that a 30 s photoactivation time dosage was enough to recover the antigenicity of HEL-K$_{96}$NPE. Extended photoactivation time (such as 300 s) did not further increase the antigenicity of these photocaged HEL variants.

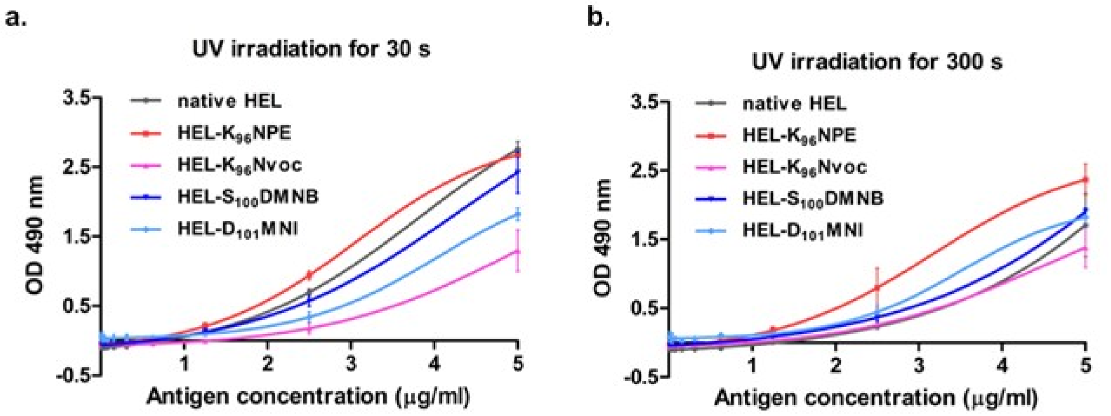

Figure S18. Binding capacity of HyHEL-10 antibody to HEL and four photocaged HEL variants was detected by ELISA. Shown are binding curves of HyHEL-10 antibody with each type of HEL coated on ELISA plate in a 1:2 serially diluted manner starting from 5 μg/mL. Each HEL variant had been irradiated under 365 nm UV light (18 mW/cm^2) for (a) 30 s or (b) 300 s respectively.

Each line represents three independent ELISA. Bars represent mean ± SEM.

3. Photolysis kinetics of [Cys[94]-Lys(NPE)[96]-Leu[129]] and [Cys[94]-Lys(Nvoc)[96]-Leu[129]]. The photolysis kinetics of [Cys[94]-Lys(NPE)[96]-Leu[129]] (K_{96}NPE) and [Cys[94]-Lys(Nvoc)[96]-Leu[129]] (K_{96}Nvoc) was examined upon exposure to UV light (365 nm, 18 mW/cm^2) for the times indicated (0, 5, 10, 15, 20, 30, 40, 50 or 60 s). We used HPLC to quantify the uncaging efficiency of these two peptides by examining the percentage of photolytic peptides in the total peptides population. It suggested that significantly more UV light exposure time (up to 600 s) was needed to achieve quantitative conversion for K_{96}Nvoc. Analytical HPLC traces for the removal of NPE caging group from K_{96}NPE after irradiation for 0 s, 2 s, 5 s, 10 s, 20 s, 30 s, and 60 s were provided in Figure S19.

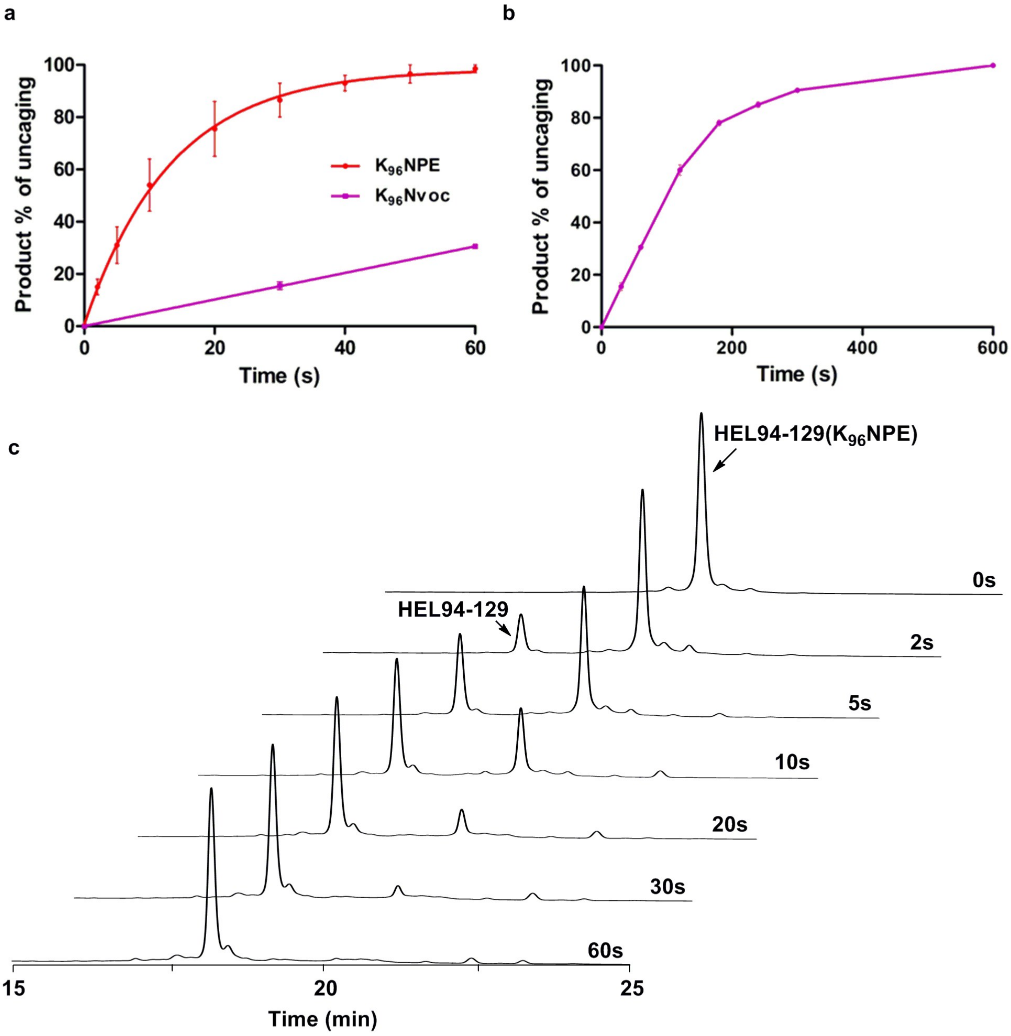

Figure S19. Photolysis kinetics of K_{96}NPE and K_{96}Nvoc. The photolysis of K_{96}NPE and K_{96}Nvoc were studied via HPLC analysis by submitting a solution of these peptides (1 mg/mL) to various durations of exposure to 365 nm UV light (18 mW/cm^2). The percentage of each was plotted versus time of irradiation for (a) both within 60 s, and (b) K_{96}Nvoc within 600 s. The mean of two experiments was plotted for both (a) and (b), and the standard deviation was represented by error bars. (c) Analytical HPLC traces for the removal of NPE caging group after irradiation for 0 s, 2 s,

5 s, 10 s, 20 s, 30 s, and 60 s. Chromatographic separations were performed by using a linear gradient (20-50%) of buffer B in buffer A over 20 min after an initial isocratic phase of 1% buffer B in buffer A for 2 min on a C18 column with a flow rate of 1 mL/min.

4. The binding affinity of synthesized HEL and four photocaged HEL variants to HyHEL-10 antibody tested by SPR. We analyzed the binding affinity (K_D), association (K_a) and dissociation (K_d) kinetics of each type of HEL species toward HyHEL-10 antibody by surface plasmon resonance (SPR) (Biacore T200). HyHEL-10 was immobilized onto the gold surface of a CM5 sensor chip as a ligand. The conditions used for each analyte were listed in Table S3. Each analyte was tested three times. The results were shown in Figure S20. The binding of HEL to HyHEL-10 had a K_D value of 9 pM, K_a of $(6.3 \pm 2.1) \times 10^6$ ($M^{-1} \cdot S^{-1}$) and K_d of $(5.1 \pm 0.6) \times 10^{-5}$ (S^{-1}), which were consistent with the reported data.[9] The binding affinity of HEL-S_{100}DMNB, HEL-D_{101}MNI, HEL-K_{96}NPE and HEL-K_{96}DMNB to HyHEL-10 was about 300-, 400-, 6800- and 6000-fold weaker compared to native HEL. For HEL-D_{101}MNI, it was caused by the extremely fast dissociation rate (about 700-fold faster than native HEL). But for HEL-S_{100}DMNB and HEL-K_{96}NPE, the slow association played a dominant role for the reduced binding affinity. Especially for HEL-K_{96}NPE, the K_a value was decreased by more than 2000-fold. Therefore, caging at Lys96 can effectively reduce the binding affinity (6000-fold weaker) of HEL toward HyHEL-10.

Table S3. The conditions used for SPR.

Analyte	HEL	HEL-K_{96}NPE	HEL-K_{96}DMNB	HEL-S_{100}DMNB	HEL-D_{101}MNI
Buffer	HBS-P	HBS-P	HBS-P	HBS-P	HBS-P
Concentration gradient (nM)	0.03125	125	125	7.5	1.25
	0.125	250	250	15	2.5
	0.5	500	500	30	5
	2	1000	1000	60	10
	8	2000	2000	120	20
Concentration repeat (nM)	0.5	500	500	30	5
Contact time (s)	180	180	180	180	180
Dissociation time (s)	600	600	600	600	300
Regeneration Buffer	Gly·HCl pH 1.5	Gly·HCl pH 1.5	Gly·HCl pH 1.5	Gly·HCl pH 1.5	Gly·HCl pH 1.5
Regeneration time (s)	60	60	60	60	60

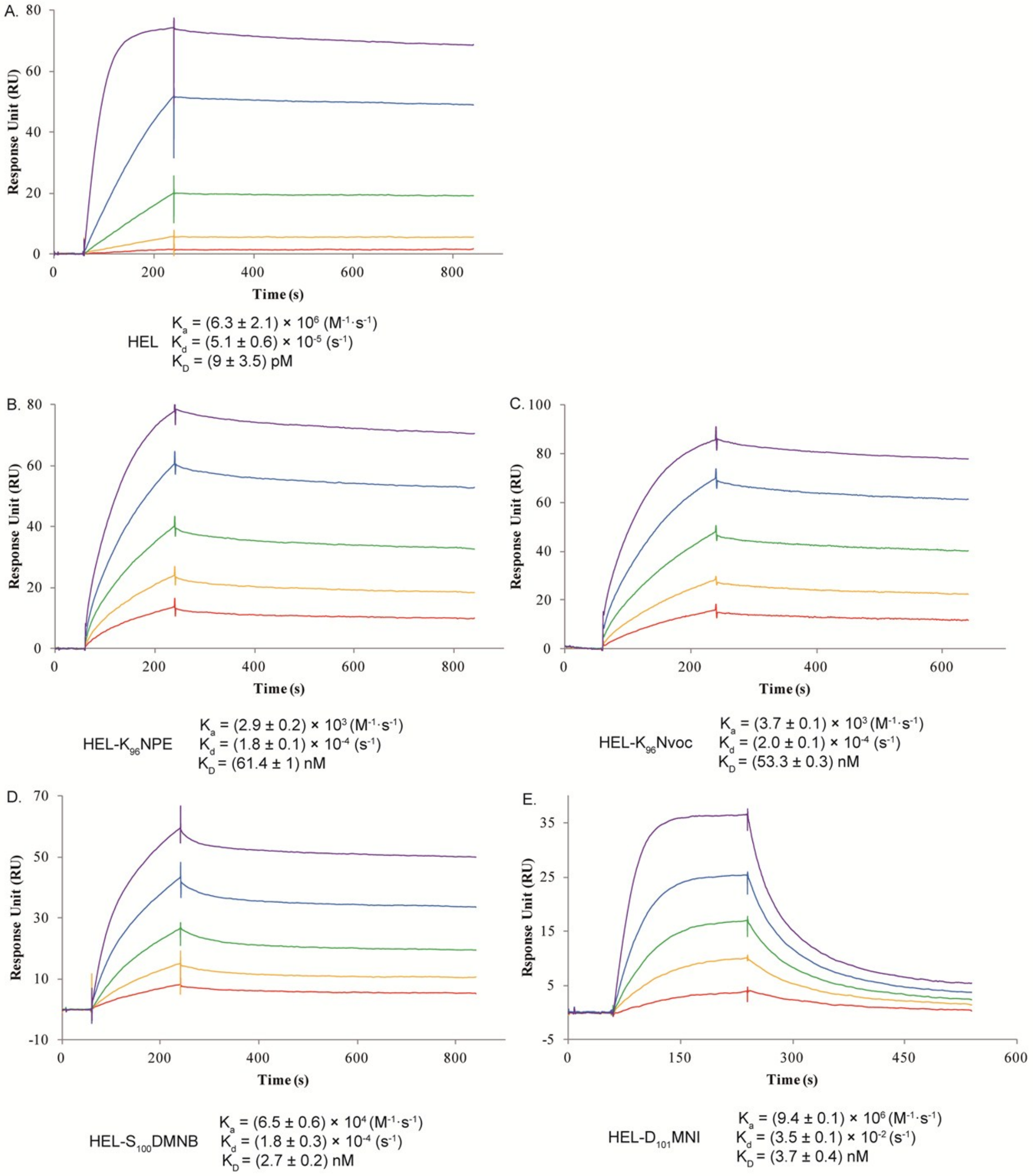

Figure S20. The affinity of HEL and four caged HEL variants to HyHEL-10 antibody tested by SPR for (A) HEL, (B) HEL-K_{96}NPE, (C) HEL-K_{96}Nvoc, (D) HEL-S_{100}DMNB, and (E) HEL-D_{101}MNI.

5. Flow Cytometry analysis for the early B cell activation marker CD86. The results of flow cytometry analysis for the B cell activation marker CD86 were shown in Figure S21. Photoactivated HEL-K_{96}NPE efficiently up-regulated the activation marker CD86 on HEL-specific primary B cells from MD4 transgenic mice the same as native HEL, while HEL-K_{96}NPE was totally inert after an incubation duration of 12 h.

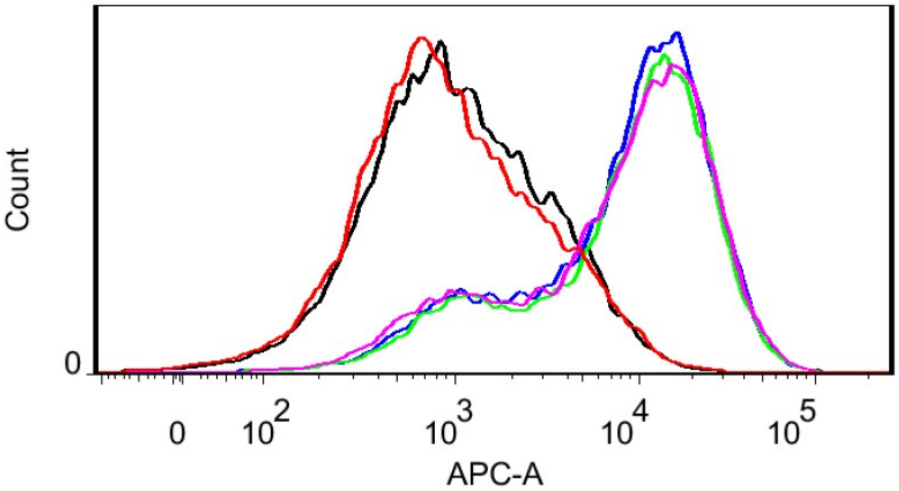

Figure S21. Photoactivated HEL-K_{96}NPE effectively up-regulated the expression of CD86 on HEL-specific primary B cells isolated from MD4 transgenic mice using flow cytometry analysis. Black: PBS (negative control); Red: HEL-K_{96}NPE without irradiation by UV light; Blue: wild type HEL; Green: wild type HEL irradiated with UV light (365 nm, 18 mW/cm^2) for 60s; Pink: HEL-K_{96}NPE irradiated with UV light (365 nm, 18 mW/cm^2) for 60s. The mean fluorescence intensity (mFI) of HEL-specific B cells activated by photoactivated HEL-K_{96}NPE was the same as HEL.

Part IV: Molecular imaging by TIRFM to quantify the formation of B cell immunological synapse

1. HEL-K$_{96}$NPE was more efficient to trigger the accumulation of BCR molecules into the B cell immunological synapse than that of HEL-K$_{96}$Nvoc under the same time dosage of 405 nm laser photoactivation. Upon photoactivation, both of them can trigger the accumulation of BCRs into the B cell immunological synapse. But the efficiency of HEL-K$_{96}$NPE was higher than that of HEL-K$_{96}$Nvoc (Figure S22). Therefore, we used HEL-K$_{96}$NPE to do all the following experiments.

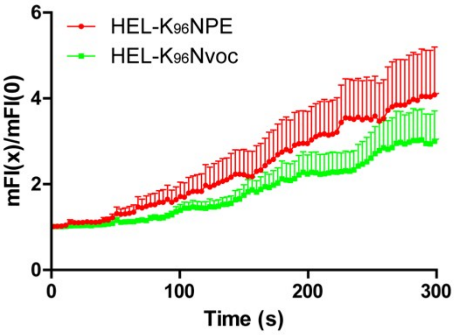

Figure S22. Photoactivation of HEL-K$_{96}$NPE or HEL-K$_{96}$Nvoc by 405 nm laser triggered the accumulation of BCRs into the B cell immunological synapse. The green line represented the responses of B cells triggered by photoactivated HEL-K$_{96}$Nvoc, and the red line represented that of HEL-K$_{96}$NPE. Normalized data was collected from 20 cells. Bars represent mean ± SEM.

2. By-products during the photolysis of NPE cannot trigger B cell responses. A control experiment by immobilizing peptide [Cys^{94}-Lys(NPE)^{96}-Leu^{129}] on the surface of coverslips was done to exclude the possibility that the observed responses of the accumulation of BCRs into B cell immunological synapse were induced by the by-products from photolysis, such as *o*-nitrosoacetopheneone. Under the same photoactivation conditions (405 nm laser for 30s at the TIRF imaging mode), we observed that the mFI of HyHEL-10 BCRs within the B cell immunological synapse increased slightly by about 20%, which was much weaker compared to the 400% increase caused by the photoactivation of HEL-K$_{96}$NPE (Figure S23).

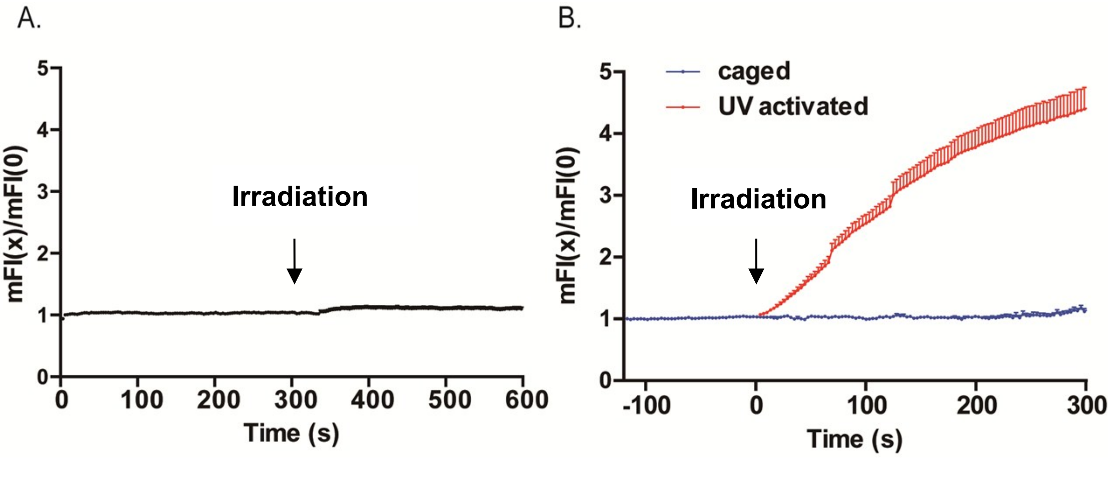

Figure S23. Irradiation of [Cys^{94}-Lys(NPE)^{96}-Leu^{129}] by 405 nm laser did not drive the accumulation of BCRs into the B cell immunological synapse. The responses of B cells triggered by photoactivated (A) [Cys^{94}-Lys(NPE)^{96}-Leu^{129}] and (B) HEL-K_{96}NPE (The red line). Normalized data was collected from 20 cells. Bars represent mean ± SEM.

3. Photocaged HEL-K_{96}NPE is much more amenable a tool to capture the early events in the formation of B cell immunological synapse than HEL.

3.1 The response of B cells encountering HEL coated on the coverslips by TIRF imaging. Because HEL is fully antigenic, when HEL-specific primary B cells from MD4 transgenic mice were loaded to the coverslips, the HyHEL-10 BCRs will immediately recognize HEL and initiate the adhesion and spreading responses and the subsequent accumulation of BCRs into the B cell's contact with the coverslips. All these events can be initiated in just a few seconds, making it difficult to separate each of these events in TIRF imaging. Indeed, when working with HEL antigen, we can still observe the accumulation of BCRs into the B cell's contact with the coverslips, but we can only use a subjective time point as the time ZERO and did the normalization for data analysis (Figure S24). However, there was significant higher BCR fluorescent intensity within the B cell's contact with the coverslips, suggesting the onset of BCR accumulation, compared to the B cells on antigen-free coverslips.

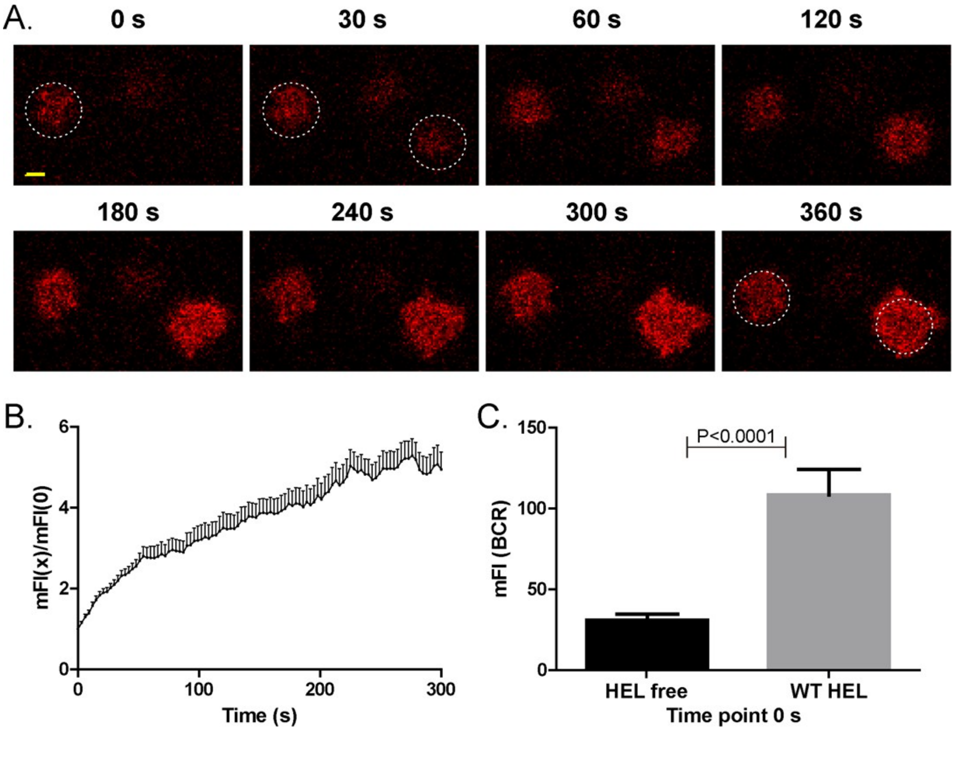

Figure S24. The responses of B cells encountering HEL coated coverslips by TIRF imaging. (A) Representative TIRF images of HEL-specific primary B cells from MD4 transgenic mice in contact with HEL at the indicated time points. Dashed white color circle indicated the border of two independent cells within the TIRF images. Scale bar is 1.6 μm. (B) The mFI of BCR within the immunological synapse. (C) The statistical analysis of mFI of BCR in B cells loaded on HEL-free coverslips for 300 s or B cells encountering HEL at the time ZERO. Bars represent mean ±

SEM, normalized data was collected from 20 cells.

3.2 No obvious responses of B cells encountering HEL-K_{96}NPE without photoactivation. The representative still images for the responses of the HEL-specific primary B cells in contact with HEL-K_{96}NPE at the indicated time points without photoactivation was shown in Figure S25. The results indicating that there were no obvious accumulations of the BCRs into the B cell's contact with the coverslips in the 300 s TIRF imaging time course if there was no 405 nm laser based photoactivation.

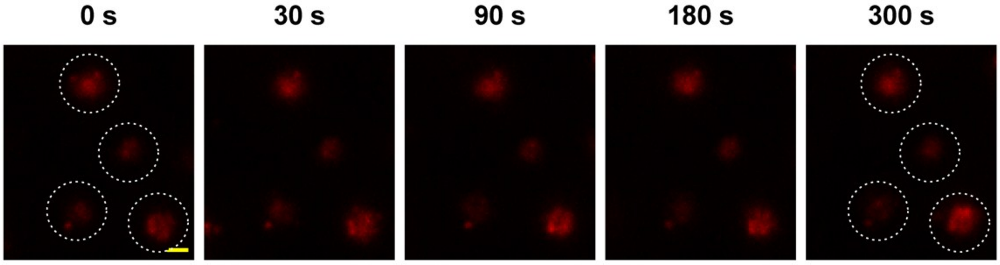

Figure S25. No obvious responses of HEL-specific primary B cells encountering the HEL-K_{96}NPE without photoactivation as examined by TIRF imaging. Representative TIRF images of HEL-specific primary B cells from MD4 transgenic mice in contact with HEL-K_{96}NPE at the indicated time points. Dashed white color circle indicated the border of four independent cells within the TIRF images. Scale bar represents 1.5 μm.

3.3 Only the B cells in the photoactivated region can be triggered for the responses of accumulating BCRs into the B cell immunological synapse. We used a defined region of interest (ROI) (195.8 μm^2) with the smallest TIRF illumination area in our TIRF microscope to do the photoactivation. When doing the photoactivation, only the 195.8 μm^2 sized ROI was illuminated by 405 nm photons. After the photoactivation, we immediately restored the size of TIRF imaging plane to 5898.2 μm^2. By doing so, we can image the synaptic accumulation of BCRs from B cells within the photoactivated area, as a side by side control we can also image the accumulation of BCRs from B cells that were far beyond the ROI in the same TIRF imaging plane. The results showed that only B cells within the ROI but not the B cells sitting far beyond the activated ROI showed the synaptic accumulation of BCRs, suggesting that B cell receptor only accumulated at the activated regions due to high antigenic HEL concentration at the interface. The results were shown in Figure S26.

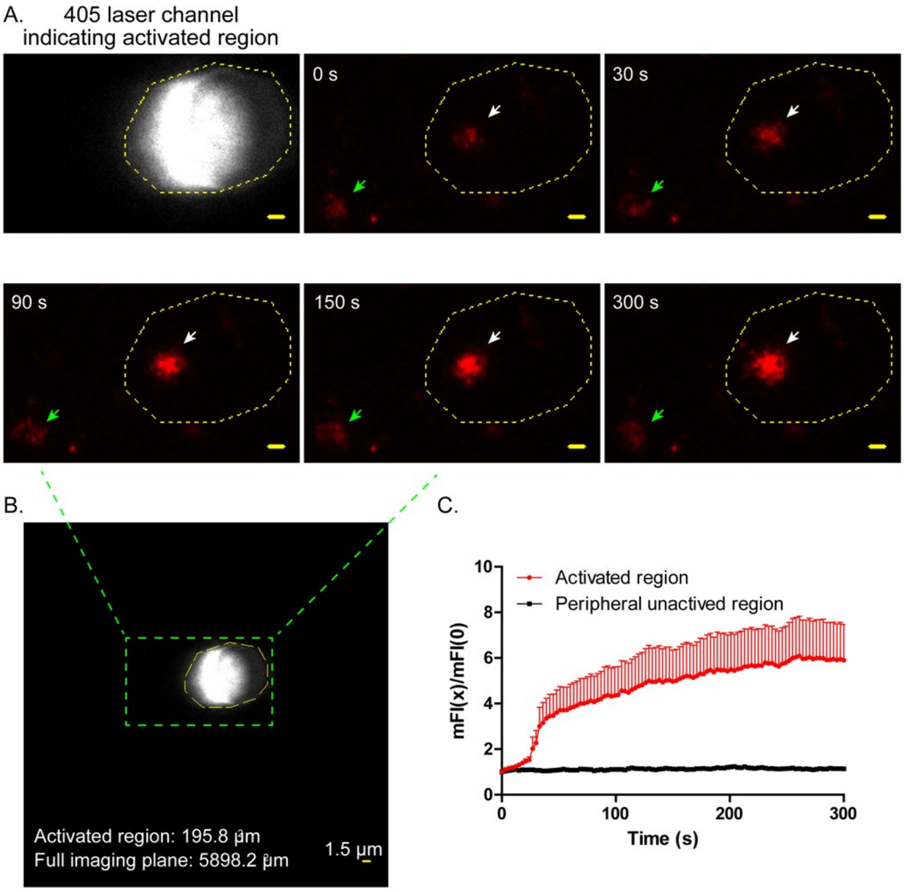

Figure S26. Only the B cells in the photoactivated region can be triggered for the responses of accumulating BCRs into the B cell immunological synapse. (A) The region of interest (ROI as indicated by the dashed line in yellow color) was defined as the smallest TIRF illumination area in TIRF microscope. Only the HEL-K_{96}NPE inside the ROI can be photoactivated by 405 nm photons. TIRF imaging showed that only B cells (as indicated by the white colored arrow) within the ROI but not the B cells (as indicated by the green colored arrow) sitting far beyond the activated ROI showed the synaptic accumulation of BCRs. Scale bar represents 1.5 µm. (B) A representative images showing the position of the photoactivated region (indicated by the dashed line in yellow color) and the full TIRF imaging plane of the TIRF microscope. The dashed line in green color demonstrated sub-region in the full TIRF imaging plane that was highlighted and amplified in (A). (C) The statistical comparison of the accumulation of BCRs within the B cell immunological synapse within the photoactivated ROI (red colored line) versus the control B cells within the region far beyond the photoactivated ROI (black colored line). Bars represent mean ± SEM.

4. The accumulation of BCRs into the B cell immunological synapse is dependent on the dosage of 405 nm photons in a linear manner. We performed the light-dose dependent experiment to photoactivate HEL-K_{96}NPE by using 0%, 0.5%, 2%, 5%, 10%, 20%, 30%, 40% and 50% of the 405 nm laser output power. At 50% output laser power, we observed severe photobleaching of the Alexa Fluor 647 conjugated Fab fragment of anti-mouse IgM antibodies that were used to label IgM-BCRs on the surface of MD4 primary B cells. Thus, we did not use much higher output laser powers. We observed no responses of B cells using light dosage between

0% and 0.5%, suggesting the existence of a triggering threshold. Thus, we focused on the 405 nm laser output power from 2% to 40% in the light dose experiment, which showed that the synaptic accumulation of BCRs was obvious dependent on the dosage of 405 nm photons in a linear manner (Figure S27).

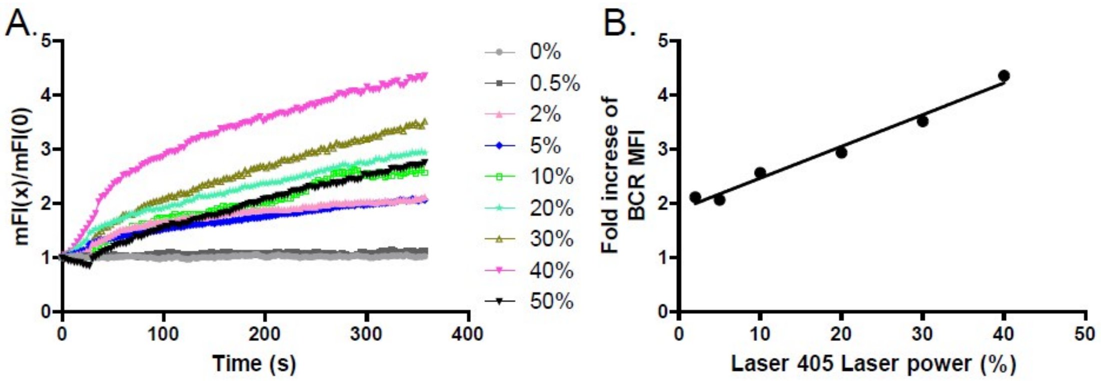

Figure S27. The accumulation of BCRs into the B cell immunological synapse is dependent on the light dosage of 405 nm photons in a linear manner. (A) Time lapse TIRF imaging showing the dynamics of the accumulation of BCR within the B cell immunological synapse upon the photoactivation of HEL-K_{96}NPE by different light-dosage of 405 nm laser. Normalized data was collected from 20 cells for each group. (B) The fold increase of mFI of the synaptic accumulated BCRs at the time point of 360 s was obvious dependent on the dosage of 405 nm photons in a linear manner. The corresponded incident 405 laser density at each of the laser output power was 0.13 W/cm^2 (2%), 0.18 W/cm^2 (5%), 0.52 W/cm^2 (10%), 2.0 W/cm^2 (20%), 4.4 W/cm^2 (30%), and 7.3 W/cm^2 (40%).

5. Mathematical quantification of BCR microclusters for fluorescence intensity (FI). The FI of the microclusters upon 2D-Gaussian fitting were used to quantify the BCR microclusters and created the 2.5D Gaussian images (Figure S28).

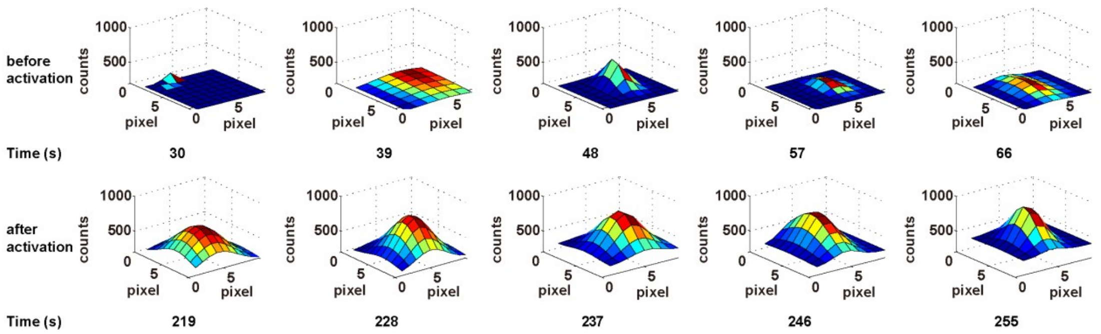

Figure S28. Photocaged HEL-K_{96}NPE drives the formation of prominent BCR microclusters upon the photoactivation of 30 s by a 405 laser. Shown were 2.5D Gaussian images of representative BCR microclusters before (top panel) and after (bottom panel) UV exposure. The clusters located in the same position of images at each indicated time point.

6. Mathematical quantification of the size of B cell's contact with the coverslips presenting HEL-K_{96}NPE before and after photoactivation.

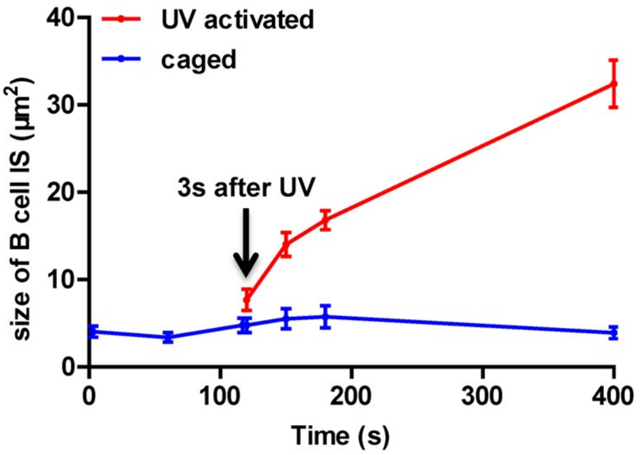

Figure S29. Photocaged HEL-K_{96}NPE dramatically increases the size of B cell immunological synapse as soon as 3s after the photoactivation by 405 nm laser. Shown was the size of the contact area (B cell immunological synapse) of MD4 primary B cells that were loaded on cover slides coated with HEL-K_{96}NPE before (blue) and after (red) 30 s of 405 nm laser irradiation. Bars represent mean ± SEM, data were collected from 10 single MD4 B cells.

7. Wild type HEL induced calcium oscillation at the single cell level. The wild type HEL induced oscillating calcium signaling with irregular oscillating cycle (Figure S30). The behaviors of the same B cell in the quiescent state before engaging native HEL cannot be captured.

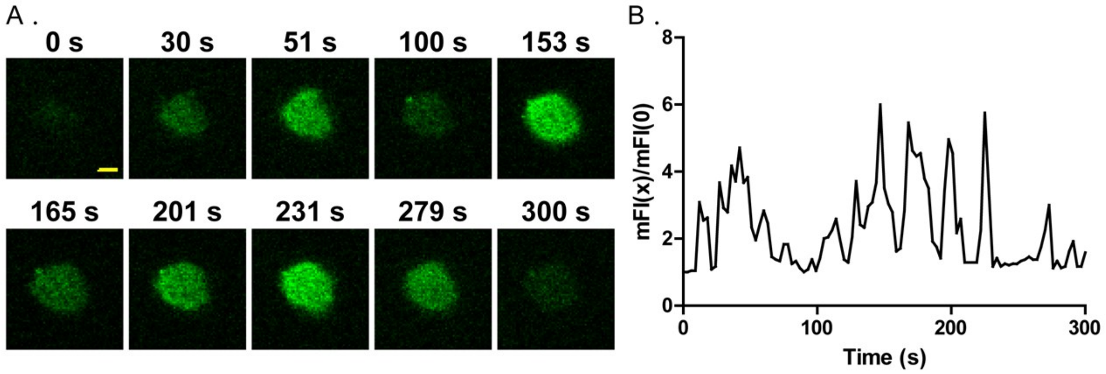

Figure S30. Wild type HEL induced calcium oscillation at the single cell level. (A) Representative TIRF images and (B) normalized mFI of single cell calcium oscillation. Scale bar, 1.6 μm.

Part V: Supporting movie legends

Supporting Movie 1. 405 nm laser photoactivation efficiently recovered the antigenicity of HEL-K_{96}NPE and MD4 primary B cells started to show the spreading responses and the significant recruitment of BCR microclusters within the B cell immunological synapse.

MD4 primary B cells were added to a glass surface pre-coated with 10 μg/mL HEL-K_{96}NPE. HyHEL-10 BCRs were pre-labeled with Alexa Fluor 647-conjugated Fab fragment Goat anti-mouse IgM. The responses of HyHEL-10-BCRs on the plasma membrane of MD4 primary B cells were imaged by TIRFM. The imaging time is 8 min (156 frames with 3.2 s intervals when imaging), and the video is shown at 33 frames per second (FPS). The full chip red colored flash in the movie represents the 30 s time frame of a 405 nm laser photoactivation.

Supporting Movie 2. HEL-K_{96}NPE efficiently evoked calcium oscillation of MD4 primary B cells after a 30 s photoactivation by a 405 nm laser.

MD4 primary B cells were loaded to a glass surface pre-coated with 10 μg/mL HEL-K_{96}NPE. These cells are pre-stained with Fluo-4 for calcium imaging. The imaging time is 8 min (151 frames with 3.2 s intervals when imaging), and the video is shown at 33 FPS. Shown are the calcium influx responses of MD4 primary B cells when encountering HEL-K_{96}NPE without (left panel) or with (right panel) the photoactivation by a 405 nm laser.

Part VI: Supporting references

1. J.-S. Zheng, S. Tang, Y.-K. Qi, Z.-P. Wang, L. Liu, *Nat. Protoc.* **2013**, *8*, 2483-2495.

2. W. Liu, T. Meckel, P. Tolar, H. W. Sohn, S. K. Pierce, *Immunity* **2010**, *32*, 778-789.

3. W. Liu, T. Meckel, P. Tolar, H. W. Sohn, S. K. Pierce, *J. Exp. Med.* **2010**, *207*, 1095-1111.

4. Z. Wan, S. Zhang, Y. Fan, K. Liu, F. Du, A. M. Davey, H. Zhang, W. Han, C. Xiong, W. Liu, *J. Immunol.* **2013**, *190*, 4661-4675.

5. S. Bourgault, M. Létourneau, A. Fournier, *Peptides*, **2007**, *28*, 1074-1082.

6. W. F. Veldhuyzen, Q. Nguyen, G. McMaster, D. S. Lawrence, *J. Am. Chem. Soc.* **2003**, *125*, 13358-13359.

7. M. C. Venuti, G. H. Jones, R. Alvarez, J. Bruno, *J. Med. Chem.* **1987**, *30*, 303-318.

8. S. Tang, J. Y. Cheng, J.-S. Zheng, *Tetra. Lett.* **2015**, *56*, 4582-4585.

9. K. A. Xavier, R. C. Willson, *Biophys. J.* **1998**, *74*, 2036-2045.
